# Ceramide Synthase 5 Deficiency Aggravates Dextran Sodium Sulfate-Induced Colitis and Colon Carcinogenesis and Impairs T-Cell Activation

**DOI:** 10.3390/cancers12071753

**Published:** 2020-07-01

**Authors:** Khadija El-Hindi, Sebastian Brachtendorf, Jennifer Christina Hartel, Stephanie Oertel, Kerstin Birod, Sandra Trautmann, Dominique Thomas, Thomas Ulshöfer, Andreas Weigert, Olaf Utermöhlen, Martin Krönke, Sabine Grösch

**Affiliations:** 1 Institute of Clinical Pharmacology, Faculty of Medicine, Goethe-University Frankfurt, 60590 Frankfurt, Germany; El-Hindi@med.uni-frankfurt.de (K.E.-H.); Sebastian.Brachtendorf@web.de (S.B.); jhartel@med.uni-frankfurt.de (J.C.H.); stephi1106@hotmail.com (S.O.); k.birod@med.uni-frankfurt.de (K.B.); trautmann@med.uni-frankfurt.de (S.T.); thomas@med.uni-frankfurt.de (D.T.); 2 Department of Life Sciences, Goethe-University Frankfurt, 60590 Frankfurt, Germany; 3 Fraunhofer Institute for Molecular Biology and Applied Ecology IME, Project Group Translational Medicine and Pharmacology (TMP), 60596 Frankfurt, Germany; Thomas.Ulshoefer@ime.fraunhofer.de; 4 Institute of Biochemistry I-Pathobiochemistry, Faculty of Medicine, Goethe-University Frankfurt, 60590 Frankfurt, Germany; weigert@biochem.uni-frankfurt.de; 5 Center for Molecular Medicine Cologne, Institute for Medical Microbiology, Immunology and Hygiene, University of Cologne, 50935 Cologne, Germany; olaf.utermoehlen@uk-koeln.de (O.U.); m.kroenke@uni-koeln.de (M.K.)

**Keywords:** colitis, DSS, CerS, T-cell, TEER, ceramide, colon, cancer

## Abstract

Ceramide synthase 5 is one of six enzymes that catalyze the production of ceramides from sphingosine or sphinganine. Ceramides are important components of cell membranes and act as signaling molecules. Previously it has been shown that ceramide synthase 6 and 2 influence colitis in several animal models with sometimes opposite effects. Here, we investigated the disease course of dextran sodium sulfate-induced acute colitis and azoxymethane/dextran sodium sulfate-induced colitis-associated colon cancer in mice with global ceramide synthase 5 knockout (CerS5-ko) or with ceramide synthase 5 knockout restricted to the colon epithelium (CerS5fl/fl VilCre). We monitored disease development and analyzed colon barrier function as well as the immune cell status in these mice. CerS5-ko mice but not CerS5fl/fl-VilCre mice were more susceptible to acute and chronic inflammation. However, the cell barrier function of colon epithelial cells was not disturbed by downregulation of ceramide synthase 5. Instead, untreated CerS5-ko mice displayed reduced numbers of CD3^+^ immune cells in the spleen, colon, and blood, especially of intraepithelial CD8^+^ T-cells, which was not obvious in CerS5fl/fl Vil Cre mice. Reduced T-cell number in colon tissue of CerS5-ko mice was accompanied by a reduced expression of IL-1β, IFNγ, and IL-4. In vitro investigations revealed that knockdown of ceramide synthase 5 in T-cells impaired T-cell activation. In summary, we show that CerS5-ko mice were more susceptible to dextran sodium sulfate-induced colitis and azoxymethane/dextran sodium sulfate-induced colitis-associated colon cancer. A reduced number of T-cells in the colon epithelium that was already the case in untreated CerS5-ko mice might have contributed to this effect.

## 1. Introduction

Ulcerative colitis (UC) is a chronic disease that is characterized by diffuse inflammation of the rectal and colonic mucosa. Ongoing chronic inflammation predisposes colitis patients to an enhanced risk of colitis-associated colon cancer (CAC), which increases with time. The incidence rates of CAC at 10, 20, and 30 years after diagnosis of UC in te latest studies were 0.91, 4.07, and 4.55 (measured in cases per 1000 patient year) [1]. The drivers of inflammation-associated cancer are manifold, comprising genetic mutations, changes in the microbiome, and nutrition such as the “Western diet”, which is more pro-inflammatory, whereas a high fiber-diet has a more positive impact [2]. The positive effect of a high-fiber diet is ascribed to short-chain fatty acids, such as acetate, butyrate, and propionate, which are produced by the microbiome during the fermentation of a high-fiber diet in the colon [3,4]. Tylichova et al. investigated the effect of sodium butyrate (NaBt) in vitro and showed that NaBt alters the cellular lipidome in human colon cancer cells. Amongst others, it leads to an increase of C16:0-ceramide and enhanced ceramide synthase (CerS) 5 expression [5]. Additionally in human colitis patients, we were previously able to show that ceramides and CerS are significantly deregulated in dependency of the disease score in colon as well as in white blood cells [6]. Due to these data, we wonder how ceramide synthase 5 (CerS5) might influence colitis and CAC. 

Ceramides are important membrane lipids but also act as second messengers in cellular signaling pathways. They play an important role in cancer development and chemosensitivity [7,8]. Six human CerS are known, displaying substrate-specific production of ceramides with distinct chain lengths. CerS1 mainly synthesizes C18-Cer, CerS4 synthesizes C18-/C20-Cer, CerS5 and CerS6 mainly produce C14- and C16-Cer, whereas CerS2 synthesizes the very long-chain C22/C24-Cer, and CerS3 generates very long ceramides with chain lengths up to C34. It has already been shown that in a dextran sulfate sodium (DSS)-induced inflammation mouse model, CerS6 deficiency enhances inflammation [9]. However, the adoptive transfer of CerS6-deficient splenocytes into recombination activating gene 1 (RAG-1)-deficient recipients resulted in a reduced intestinal inflammation score in comparison to mice receiving CerS6-wt splenocytes [10]. In addition, in CerS2-deficient mice, DSS-induced colitis and CAC are enhanced in comparison to wt mice [11]. In the intestine, CerS2, CerS4, CerS5, and CerS6 are predominantly expressed [12]. Although CerS5 and CerS6 have the same substrate specificity, mice with a knockdown of these enzymes show distinct differences under pathophysiological conditions [13]. In colon cancer development, several sphingolipid metabolizing enzymes are deregulated [14,15], but the molecular mechanisms are largely unknown. Therefore, we analyzed the impact of CerS5 deficiency in the acute and chronic DSS model as well as on CAC. To investigate acute colitis and CAC in mice, we chose dextran sulfate sodium (DSS) single treatment (which induces acute inflammation), or repeated treatment of mice with DSS in combination with azoxymethane (AOM) to induce CAC. Oral administration of DSS in mice resembles several features of human UC, such as weight loss, bloody diarrhea, and ulcer formation, and is therefore a common model for UC [16]. Additionally, as a chronic model, it also resembles the basics of CAC such as repeated epithelial damage and wound repair, which itself is a risk factor for colon cancer. AOM is used as a trigger substance to accelerate tumor development.

## 2. Results

### 2.1. CerS5-Deficient Mice Were More Susceptible to DSS-Induced Acute Colitis

**Figure 1:**
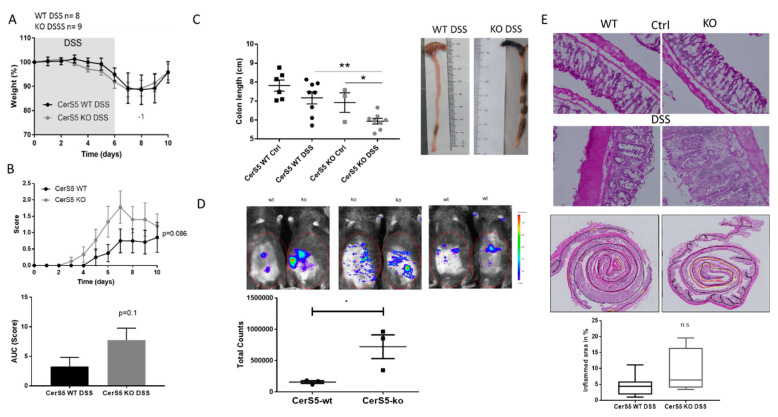
Clinical course and pathology of dextran sulfate sodium (DSS)-induced acute colitis in ceramide synthase 5 (CerS5)-wt and CerS5-knockout (ko) mice. Application of 2% DSS in the drinking water for 6 days reduced body weight (**A**) and increased disease score (**B**) of wt and CerS5-ko mice over time. Area under the curve (AUC) was calculated with the trapezoidal rule. (**C**) Colon length was significantly reduced in CerS5-wt and CerS5-ko mice after DSS treatment in comparison to control (Ctrl) mice. (**D**) Bioluminescence imaging of inflammation with an IVIS Lumina Spectrum. At day 10 of DSS treatment, colitis was detected in living animals by intraperitoneal (i.p.) injection of 200 mg/kg inflammation probe and subsequent detection of bioluminescence in the abdomen of CerS5-wt and CerS5-ko mice. The total luminescent counts in the abdominal regions of interest were quantified. (**E**) Sections of colon rolls, 10 μm in size, were stained with hematoxylin/eosin, and images were taken with a Keyence BZ-9000 microscope. Inflamed colon areas (yellow marked area in the colon rolls) were sized and related to the whole colon length (black marked area) (wt DSS group *n* = 9; ko DSS group *n* = 4). Data are mean ± standard error of the mean (SEM) of *n* = 8/9 animals, for bioluminescence imaging *n* = 3 animals in each group. Statistical analysis was performed by unpaired *t*-test (**B**–**E**) or one-way ANOVA (**C**); *p* * < 0.05, *p* ** < 0.01.

We treated CerS5-knockout (ko; global CerS5−/−) and CerS5-wt mice with 2% DSS for 6 days. CerS5-ko and -wt mice showed no differences in weight loss, but CerS5-ko mice displayed a tendency towards a higher disease score (Figure 1A,B). Moreover, colons were significantly shorter in CerS5-ko mice than in -wt mice after DSS treatment (Figure 1C) and in vivo imaging of living mice confirmed a stronger bowel inflammation in CerS5-ko mice (Figure 1D). This was also reflected in the histological score (Figure 1E). Colon inflammation in CerS5-ko mice was severe, shown as loss of intestinal crypt cells and enhanced infiltration of immune cells in the intestinal mucosa. When we analyzed the inflamed area of the whole colon, we also observed a tendency towards a severe disease state in CerS5-ko mice (Figure 1E). In summary, our data show that DSS-induced acute inflammation is more severe in CerS5-ko mice than in CerS5-wt mice. 

### 2.2. CerS5-ko Increased Tumor Development in the AOM/DSS CAC Model

**Figure 2:**
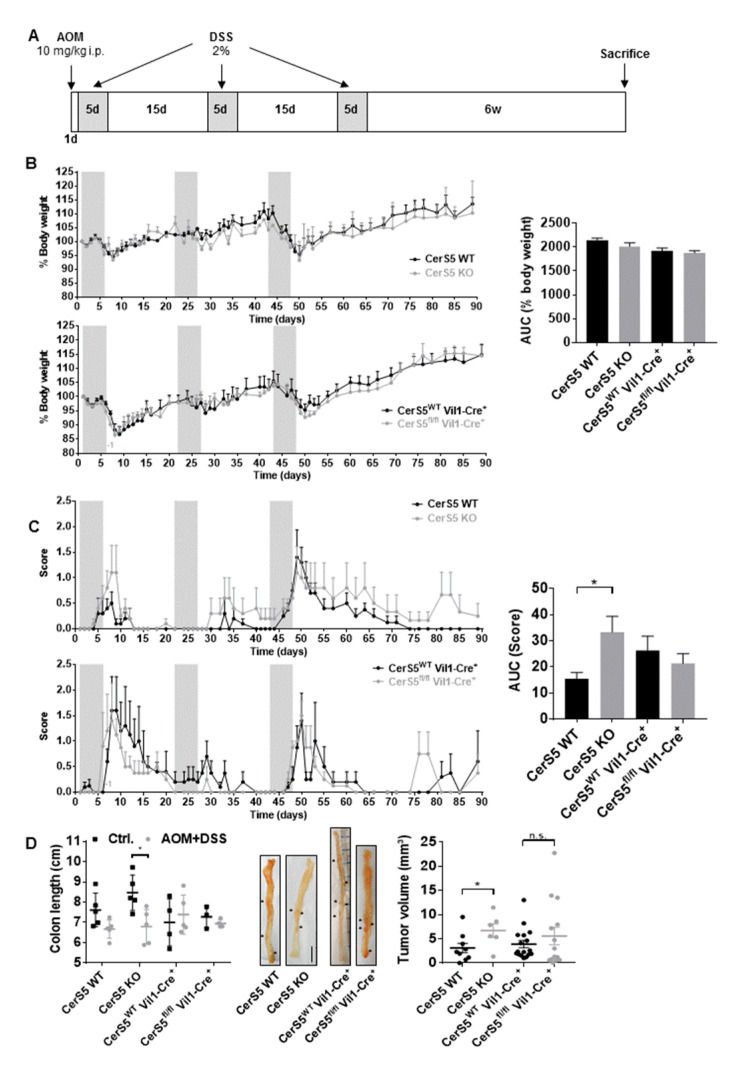
In CerS5-wt and CerS5-ko as well as in CerS5-wt-VilCre and CerS5fl/fl VilCre mice, the clinical course and pathology of AOM/DSS-induced colitis-associated colon cancer were investigated. (**A**) Treatment schemata: at day 1, 10 mg/kg AOM were injected intraperitoneally (i.p.), followed by application of three cycles of 2% DSS in the drinking water for 5 days and 15 days recovery. Body weight (**B**) and disease score (**C**) were followed up for 90 days. The area under the curve (AUC) was calculated for each treatment group and compared by Student’s *t*-test; *p* * = 0.05. At the end, mice were sacrificed and colon length and tumor development were investigated (**D**). Data are mean ± SEM. Statistical analysis was performed by one-way ANOVA. Mice *n* = 5 for each group.

In addition to the acute DSS mouse model, we investigated colon tumor development in CerS5-wt and CerS5-ko mice, as well as in mice with CerS5 ablation restricted to colon epithelial cells (CerS5fl/fl/VilCre) using the AOM/DSS mouse model. Body weight did not significantly differ during the whole observation time between CerS5-wt versus CerS5-ko and CerS5-wt/-fl/fl/VilCre mice (Figure 2B). However, the disease score in CerS5-ko mice was significantly enhanced in comparison to CerS5-wt mice (Figure 2C), indicating that CerS5-ko mice are more sensitive to chronic DSS treatment. No significant differences in body weight or disease score was observed between CerS5-wt/VilCre or CerS5fl/fl/VilCre mice. After 3 months, we analyzed colon tumor occurrence. CerS5-ko mice showed a significantly reduced colon length and larger colon tumors than CerS5-wt mice (Figure 2D and Figure S1), whereas neither colon length nor tumor volume significantly differed between AOM/DSS-treated CerS5wt/VilCre or CerS5fl/fl/VilCre mice (Figure 2D).

### 2.3. Sphingolipid Status in CerS5-ko Mice after DSS Treatment

**Figure 3:**
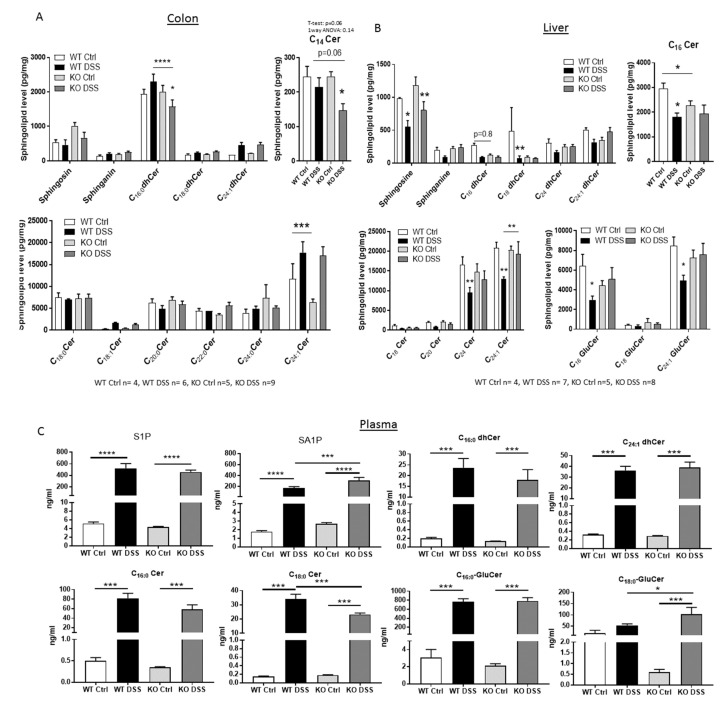
Sphingolipid levels in picogram per milligram tissue of colon (**A**) and liver (**B**) and nanogram per milliliter in plasma (**C**). Data are mean of *n* = 4–9 ± SEM; statistical analysis was performed by one-way ANOVA. *p* * = 0.05, *p* ** = 0.01, *p* *** = 0.001, *p* **** = 0.0001.

In order to prove if CerS5 knockdown has an influence on the sphingolipid level in different tissues after DSS treatment, we quantified various sphingolipids in colon, liver, and plasma from CerS5-wt and CerS5-ko mice by LC–MS/MS (Figure 3). In accordance with the already published sphingolipid data from these mice [13], we observed a significant reduction of C16:0-Cer in the liver of CerS5-ko control mice in comparison to CerS5-wt control mice (Figure 3). In the liver, CerS5 mRNA is more highly expressed than CerS6 [17]. Thus, knockdown of CerS5 resulted in a decrease of long-chain ceramide level in this tissue. In colon tissue of untreated CerS5-ko mice, the level of C16:0-dihydroceramide (dhCer) as well as of C14:0-C16:0-ceramides (Cer) did not differ in comparison to CerS5-wt mice. This might be related to an abundant expression of CerS6 in colon tissue [18], which can compensate for the production of long-chain ceramides in this tissue. However, after DSS treatment, C16:0-dhCer and C14:0-Cer levels significantly decreased only in CerS5-ko mice, indicating that after stress stimuli, the loss of CerS5 could not be compensated by CerS6. In liver tissue, very long-chain ceramides and glucosylceramides (GluCer) significantly decreased only in wt mice after DSS treatment (Figure 3). In plasma of CerS5-ko and CerS5-wt mice, the levels of several sphingolipids were significantly elevated after DSS treatment, including sphingosine-1-phosphate (S1P), sphinganine-1-phosphate (SA1P), C16:0-dhCer, C24:1-dhCer, C16:0- and C18:0-Cer, and C16:0-GluCer. However, only SA1P, C18:0-Cer, and C18:0-GluCer plasma levels significantly differed in DSS-treated CerS5-ko mice in comparison to DSS-treated wt mice (Figure 3C). 

### 2.4. Increased Sensitivity of CerS5-ko Mice to DSS-Induced Colitis Was Not Based on Defects in Barrier Function

**Figure 4:**
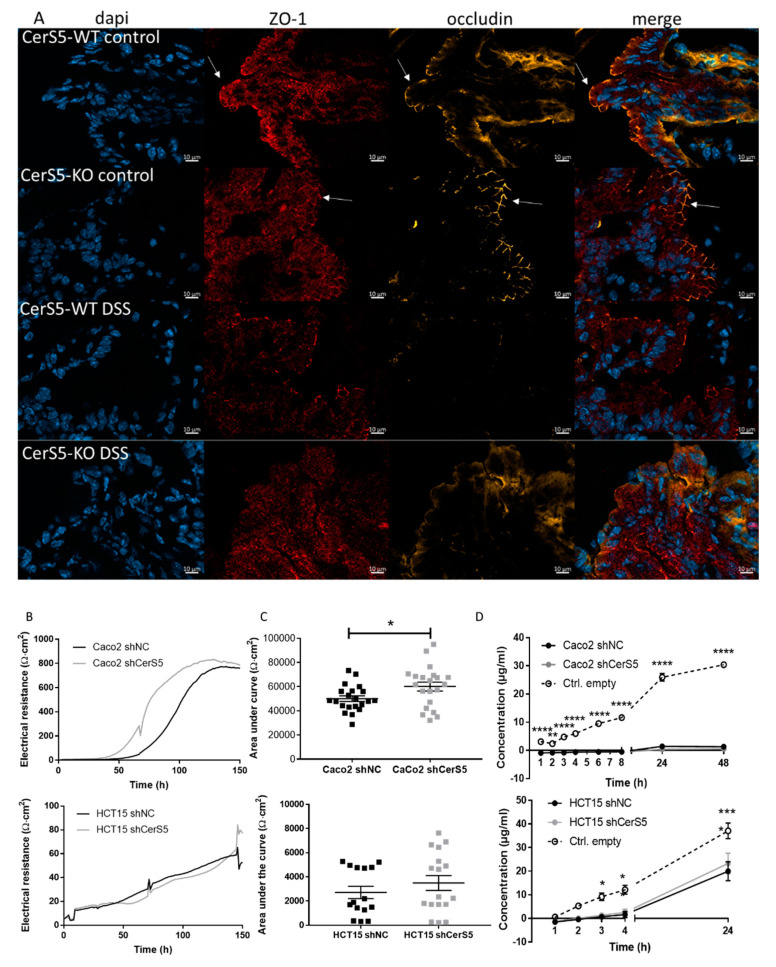
Influence of CerS5 on colon epithelial barrier. (**A**) Immunohistochemical staining of occludin and zonula occludens (ZO-1) in colon tissue from CerS5-wt and CerS5-ko mice. Slices, 10 μM in size, were fixed with Paraformaldehyde (PFA) and stained with anti-ZO-1 antibody and secondary Alexa Fluor 647-anti-rabbit antibody, anti-occludin-antibody and secondary Cy3-anti-mouse antibody, and 4′,6-diamidino-2′-phenylindoldihydrochloride (DAPI). Transepithelial electrical resistance (TEER) (**A**,**B**) and permeability assay (**C**) of Caco-2 and HCT-15 cells using a cellZscope instrument. For TEER measurement, the cells were stimulated with 1 Hz to 100 kHz and the impedance was measured each hour. The calculation of the capacity and the electrical resistance was performed by the CellZscope software. (**A**) represents an exemplary measurement of the electrical resistance of Caco-2 and HCT-15 cells and their CerS5-downregulated counterparts. (**B**) shows the mean ± SEM AUC of the electrical resistance curves of *n* = 3–4 independent experiments. Statistical analysis was performed by Student’s *t*-test; *p* * = 0.05. (**C**) Permeability of the cells was analyzed using fluorescein isothiocyanate (FITC)-dextran. FITC at 100 μg/mL (average molecular weight: 40,000 by Sigma Aldrich) were added to the upper insert when cells reached a plateau and the impedance was on a saturated and constant level. We took 20 μL from the apical medium and 66.7 μL from the lateral medium at indicated time points up to 48 h, and we measured them using the EnSpire Multimode Plate Reader (perkinElmer) at an excitation wavelength of 496 nm and an emission wavelength of 530 nm. To calculate the FITC content from the emission, we included a standard curve. Shown are mean ± SEM of *n* = 3–4 independent experiments. Statistical analysis was performed by one-way ANOVA. *p* * = 0.05, *p* ** = 0.01, *p* *** = 0.001, *p* **** = 0.0001.

Previous data in CerS2−/− mice have shown that a higher susceptibility of these mice to DSS-induced colitis and CAC was originated from a disruption of the colon barrier that was based on a decreased expression of tight junction proteins zonula occludens (ZO-1) and occludin in colon epithelial cells [11]. Therefore, we investigated the expression of occludin and ZO-1 in colon tissue of CerS5-ko and -wt mice by immunohistochemistry. Occludin and ZO-1 showed a clear apical membranous staining in colon epithelia cells of untreated CerS5-ko and CerS5-wt mice, without any obvious differences. After DSS treatment, occludin and ZO-1 diminished at the plasma membrane. In DSS treated mice, ZO-1 could be detected as patchy staining in the cytoplasm (Figure 4A). To investigate if the barrier function of colon epithelial cells is impaired by CerS5-depletion, we downregulated CerS5 expression in colon carcinoma cells by CerS5-shRNA and assessed the epithelial barrier function by determining the transepithelial electric resistance (TEER) and membrane permeability by a fluorescein isothiocyanate (FITC)-dextran permeability assay. As a cell model and for a better transfer of the results to the situation in humans, we used the human colon cancer cells Caco-2 and HCT-15 cells. Both cell lines express CerS5, which we could effectively downregulate by lentiviral transduction of CerS5-shRNA (Figure S2). In Caco-2 cells, downregulation of CerS5 enhanced TEER but had no effect on the permeability for FITC-dextran (Figure 4). Caco-2 cells form a very tight cell barrier, which was reflected by the electrical resistance reaching up to 800 Ωcm². Downregulation of CerS5 in HCT-15 cells had no effect on TEER or permeability for FITC-dextran, however, these cells formed a weaker cell barrier (max about 60 Ωm²) and were permeable for FITC-dextran (Figure 4).

### 2.5. CerS5-ko Mice Differed in Their T-cell Status

**Figure 5:**
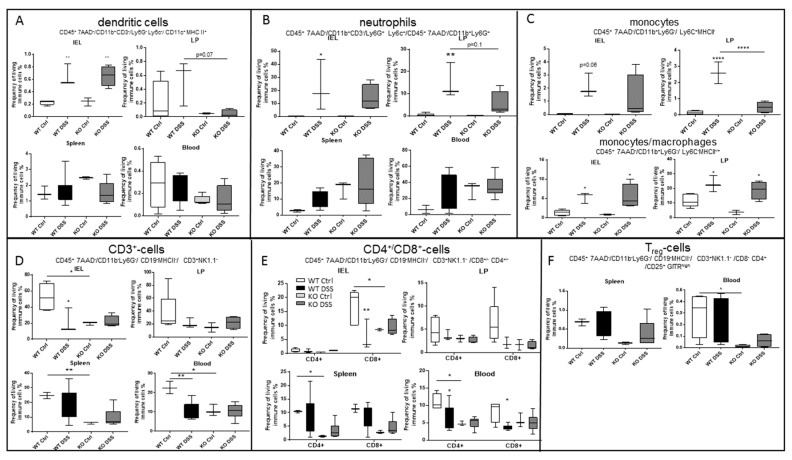

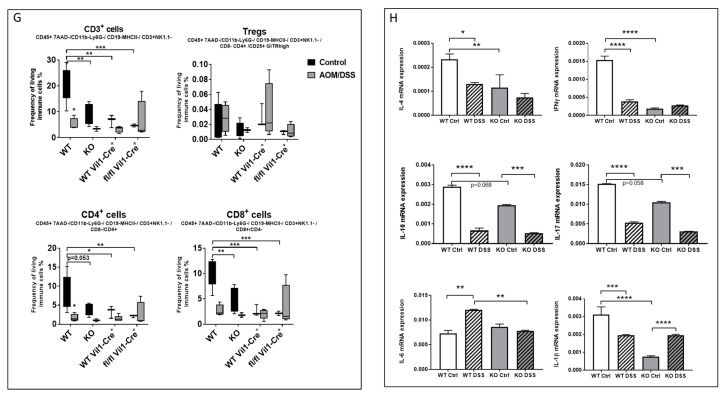
Flow cytometry analysis of different mice tissues (intraepithelial lymphocytes (IEL), lamina propria (LP), spleen, and blood) upon acute DSS treatment in CerS5-wt and -ko mice. Dendritic cells (**A**), neutrophils (**B**), and monocytes/macrophages (**C**) were increased in the colon tissue fractions after DSS treatment. By contrast, CD3^+^ T-cells were decreased in CerS5-wt mice after DSS treatment or were already low in CerS5-ko mice (control and DSS-treated) (**D**–**F**). Shown are mean ± min/max IEL: WT Ctrl *n* = 5, WT DSS *n* = 3, KO Ctrl *n* = 3, KO DSS *n* = 4; LP: WT Ctrl *n* = 5, WT DSS *n* = 3, KO Ctrl *n* = 2, KO DSS *n* = 4; spleen: WT Ctrl *n* = 3, WT DSS *n* = 6, KO Ctrl *n* = 3, KO DSS 6; blood: WT Ctrl *n* = 3, WT DSS *n* = 7, KO Ctrl *n* = 3, KO DSS *n* = 6. (**G**) Blood T-cell status in CerS5-wt and CerS5-ko mice as well as in CerS5wt-VilCre and CerS5fl/fl-VilCre mice in the chronic AOM/DSS mouse model. Blood leukocytes were isolated and quantified by flow cytometry. Here, only CD3^+^-cells are shown. Analysis was performed using FlowJo software v10. Data are mean ± SEM of *n* = 2–4 animals in each group. (**H**) Cytokine mRNA expression in colon tissue of CerS5-ko and CerS5-wt mice. RNA from colon tissue was extracted and mRNA of the various cytokines detected by real time PCR. Data are mean ± SEM of *n* = 4–6 animals in each group. Statistical analysis was performed by one-way ANOVA. *p* * = 0.05, *p* ** = 0.01, *p* *** = 0.001, *p* **** = 0.0001.

Apart from colon barrier integrity, immune cell status is also an important determinant in colitis and CAC. Therefore, we investigated the immune response of CerS5-wt and CerS5-ko mice after acute DSS treatment. Dendritic cells (DCs), neutrophils, and monocytes are the first cells that are activated in the DSS-induced colitis model and are responsible for the release of inflammatory cytokines as well as cytotoxic substances such as reactive oxygen species (ROS). After DSS treatment, we detected increased numbers of colonic DCs, neutrophils, and monocytes/macrophages in CerS5-wt and CerS5-ko mice (Figure 5). However, only the number of monocytes in the lamina propria of CerS5-ko mice were significantly reduced in comparison to CerS5-ko mice after DSS treatment. Neither the number of DCs nor neutrophils differed significantly in untreated or DSS treated CerS5-ko mice in comparison to -wt mice.

Apart from these important cellular gatekeepers of the colon, intraepithelial T-cells also contribute to homeostasis and inflammation in the gut [19]. Whereas CD4^+^ cells are mainly located in the lamina propria of the colon, T-cells with a cytotoxic profile (CD8^+^ cells) dominate in the intestinal epithelium [20]. Interestingly, in CerS5-ko control mice, CD3^+^/CD8^+^ T-cells in particular were reduced in the intraepithelial lymphocyte (IEL) fraction and CD3^+^/CD4^+^ and CD3^+^/CD8^+^ T-cells in spleen and blood in comparison to untreated wt mice (Figure 5D,E). In addition, regulatory T-cells (Treg cells) were reduced in the blood of CerS5-ko control mice in comparison to CerS5-wt control mice (Figure 5F). After DSS treatment, CD4^+^ and CD8^+^ T-cells were decreased in the blood of wt mice, which is in accordance with human data from colitis patients [20]. However, in CerS5-ko mice, we did not observe a similar decrease in CD4^+^ or CD8^+^ cells after acute DSS treatment, which might have been related to the already low amounts of T-cells in the colon, spleen, and blood of untreated CerS5-ko mice (Figure 5D,E). Additionally, in the CAC mouse model, we observed reduced numbers of blood CD3^+^/CD8^+^ cells in CerS5-ko control mice compared with CerS5-wt mice, but not in CerS5 fl/fl VilCre mice versus CerS5-wt/VilCre mice (Figure 5G). In addition to the differences in the T-cell status in colon tissue of wt and CerS5-ko mice, we measured cytokine expression of IL-1β, IFNγ, IL-4, IL-6, IL-10, and IL-17 in colon tissue. IL-1, IFNγ, and IL-4 were significantly reduced in the colon tissue of untreated CerS5-ko mice in comparison to wt mice (Figure 5H). IL-17 and IL-10 showed only a tendency to lower levels in untreated CerS5-ko mice. After DSS treatment, we observed a clear decrease in the mRNA expression level of IL-1β, IL-10, IL-17, IL-4, and IFNγ and an increase in IL-6 in CerS5-wt mice (Figure 5H), whereas in CerS5-ko mice, IL-1β increased, and IL-17 and IL-10 decreased. No changes could be observed for IL-4, IL-6, or IFNγ in CerS5-ko mice. These data clearly show that in the colon of CerS5-ko, not only T-cells were reduced, which are likely the main producers of IFNγ, IL-4, and IL-17, but also other cytokines such as IL-1β were deregulated in comparison to CerS5-wt mice.

### 2.6. CerS5 Deficiency Impaired T-cell Signaling in Human T-cells

**Figure 6:**
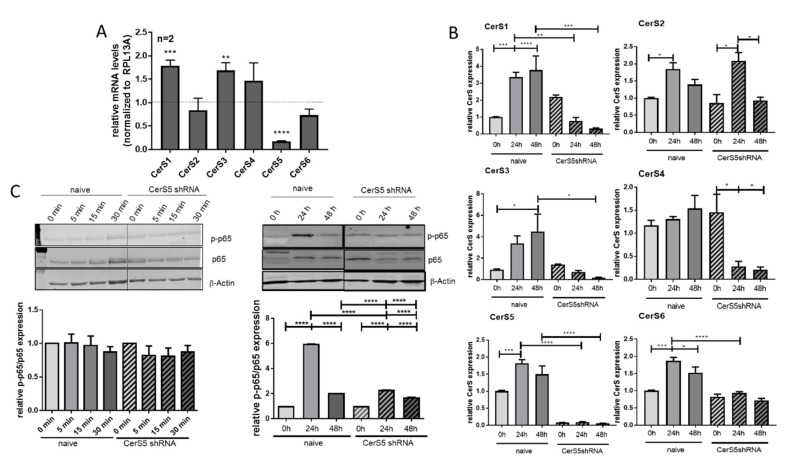
Activation of Jurkat cells is prohibited by CerS5 downregulation. (**A**) Transduction of CerS5-shRNA into Jurkat cells led to a significant reduction of CerS5 mRNA expression. (**B**) CerS mRNA expression was increased after stimulation of Jurkat cells with IL-2 (200 Units/mL) and anti-CD2/3/28 activation beads (1:1 beads-to-cell ratio) for 24–48 h. This was inhibited by CerS5 downregulation. (**C**) NF-κB activation in Jurkat cells after treatment with IL-2 (200 units/mL) and anti-CD2/3/28 activation beads (1:1 beads-to-cell ratio) for 0–48 h. NF-κB activation was detected by anti-phospho-p65 and anti-p65 antibodies by Western blot method. Data from (A) and (B) are mean ± SEM of 2–4 independent experiments measured in triplicate. (**C**) One representative blot is shown of three to five. For calculation, the expression levels of *p*-p65 and p65 were related to β-actin and subsequently related to untreated control cells. Data are mean ± SEM. Statistical analysis was performed by one-way ANOVA. *p* * = 0.05, *p* ** = 0.01, *p* *** = 0.001, *p* **** = 0.0001. The whole western blot image please find in Figure S4.

Due to the results from CerS5-ko mice, we wondered if knockdown of CerS-5 in T-cells has an influence on T-cell functions. To address this assumption, we downregulated CerS5 expression in human CD4^+^ Jurkat cells by lentiviral transduction of CerS5 shRNA (Figure 6A) and treated them with IL-2 and anti-CD2/CD3/CD28 beads for stimulation of T-cell receptor (TCR) signaling. Downregulation of CerS5 was highly significant and accompanied by an increase in CerS1 and CerS3 expression in unstimulated Jurkat cells (Figure 6A). To elucidate how CerS expression is affected by activation of Jurkat cells, we measured the expression levels of all CerS isoenzmyes 24 or 48 h after stimulation. With the exception of CerS4, expression of all other CerSs was enhanced after Jurkat cell stimulation (Figure 6B). Downregulation of CerS5 prohibited upregulation of CerS1, CerS3, CerS5, and CerS6 and significantly decreased CerS4 expression (Figure 6B). To clarify how CerS5 downregulation influences T-cell activation, we investigated NF-κB signaling by detection of phospho-p65 and p65 via Western blot. The NF-κB signaling pathway is well known to be activated after TCR stimulation [21,22] and is functional in Jurkat cells. Although, short-term treatment (5–30 min) of Jurkat cells had no effect on NF-κB activation, 24 h after treatment of Jurkat cells with IL-2/anti-CD2/CD3/CD28 beads, the level of phospho-p65 was significantly increased. In contrast, in CerS5-shRNA transduced Jurkat cells we observed a significantly reduced NF-κB activation, indicating that CerS5 is important for proper T-cell activation (Figure 6C).

## 3. Discussion

CerS5 and CerS6 are conjointly responsible for the generation of long-chain ceramides using sphingosine or sphinganine and acyl-CoAs with C14:0 or C16:0 chain lengths. Although they generate the same products, their physiological effects seem to differ significantly [13]. CerS6-ko mice are more susceptible for DSS-induced colitis than wild-type mice [9]. In our study, we observed that CerS5-ko mice were also more susceptible to DSS-induced colitis as well as to AOM/DSS-induced colitis-associated colon cancer. In general, this sensitivity can be related to an impaired colon barrier function that facilitates the invasion of colon microbes into the tissue. This mechanism seems to be most responsible for the higher susceptibility of CerS2-ko mice to DSS-induced acute colitis and CAC [11]. However, on the basis of (1) immunohistochemical staining of tight junctions proteins ZO-1 and occludin in CerS5-ko and CerS5-wt colon tissue, and (2) barrier functional tests, showing no impairment but rather an enhanced barrier function in CerS5-downregulated colon cancer cells, and 3) due to the fact that CerS5fl/fl VilCre mice showed no higher sensitivity towards AOM/DSS treatment, it seems unlikely that CerS5 has an impact on the colon barrier. Therefore, our data indicate that depletion of CerS5 in colon epithelial cells is not responsible for the higher sensitivity of CerS5–ko mice against DSS-induced colitis and CAC.

The second main mechanism contributing to an increase in the susceptibility for colitis and CAC is an imbalance in the immune system. In CerS6-ko mice, higher sensitivity towards DSS-induced colitis has mainly been referred to an increase in neutrophil infiltration [9]. In our study, we observed a significant reduction of CD^3+^ T-cells the in colon, spleen, and blood of untreated CerS5-ko mice in comparison to wt control mice. This was not seen in CerS5fl/fl VilCre mice, which let us suggest that CerS5 deficiency in lymphocytes is responsible for the reduction of T-cells. In addition to CD4+ and CD8^+^ T-cells, Treg cells were also reduced in the blood of CerS5-ko mice. These data indicate that possibly all T-cell subsets are affected by CerS5 downregulation. Until now, not much has been known about the role of CerS5 in T-cells. However, the role of CerS6 and CerS4 in T-cell function has been characterized [23]. Sofi et al. showed that CerS6-depleted CD4^+^ or CD8^+^ T-cells have a reduced expansion or migration rate and produce less IFNγ in comparison to wt counterparts [23]. This was also apparent in the adoptive T-cell transfer colitis model, where recipients of CerS6-ko T-cells developed less severe colitis and showed lower numbers of CerS6-ko T-cells in the colon than recipients of wild-type T-cells [10,23]. Additionally, in CerS5-ko mice, IFNγ as well as IL-4 was significantly reduced in untreated control mice in comparison to wt mice. Both cytokines are spontaneously produced by CD4^+^ IEL and are important for normal homeostasis of the intestinal mucosa [24]. In addition, IL-1β, IL-10, and IL-17 are important for the homeostasis in the colon and are produced by different T-cell subsets (Th17, Th1, Th2, IEL) [25]. Additionally to the reduction of CD4^+^ and CD8^+^ T-cells in colon tissue, we observed a reduced CD4^+^ T-cell number in blood and spleen in untreated CerS5-ko mice in comparison to wt mice, indicating that the T-cell population overall was affected. In CerS5fl/flVilCre, we saw no difference in CD4^+^ or CD8^+^ T-cells in the colon. Sofi et al. demonstrated that CerS6-deficient T-cells are limited in T-cell receptor (TCR) signaling, with a reduced phosphorylation of zeta-chain-associated protein kinase 70 (ZAP70) and translocation of protein kinase C theta (PKCθ) to the cytoplasma membrane [23]. In contrast, CerS4-depletion had no effect on T-cell development, but proliferation of CD4^+^ cells was enhanced, whereby proliferation of CD8^+^ cells was reduced by CerS4 knockdown [23]. Our data from Jurkat cells demonstrated that CerS5 downregulation impairs T-cell activation leading to reduced NF-κB activation. However, we could not observe an effect on cell proliferation in these cells (data not shown). Activation of Jurkat cells by anti-CD2 or -CD28 leads to an enhanced NF-κB signaling [26]. Therefore, we assume that depletion of CerS5 impairs TCR signaling in Jurkat cells. Sofi et al. were able to show that impaired TCR signaling can be restored in CerS6-ko T-cells by addition of C16:0-ceramide. Because CerS5 is also responsible for the production of C16:0-ceramide and our data demonstrated that CerS5 and CerS6 were equally upregulated after stimulation of Jurkat cells, it is possible that CerS5 and CerS6 might similarly influence TCR signaling in T-cells. In line with this, it has been shown that TCR signaling is dependent on its location into lipid raft domains that are enriched for sphingomyeline (SM 34:1,2) [27]. A reduction of C16-ceramide, as direct precursor of SM 34:1, might impair TCR signaling by destabilization of lipid raft domains. Additionally, migration of T-cells is dependent on membrane-localized receptors such as the gut-homing marker integrin α4β7 or G-protein-coupled receptor 15 (GPR15). Both are important T-cell markers that are responsible for the localization of T-cells to the colon [19,28]. It has been shown that different integrins are localized into lipid rafts [29]. As is the case for the TCR signaling, the membrane structure might be important for these receptors. Besides these two receptors, the sphingosine 1-phospate receptor 1 (S1P1) is also an important T-cell marker for T-cell egress [30] and its activation by S1P is dependent on its localization into lipid rafts [30]. Additionally S1P1 is upregulated during inflammatory bowel disease [31] and targeting this receptor either by the unspecific S1P agonist FTY720, by the specific S1P1/S1P5 agonist Ozanimod, or by the specific S1P1/S1P4/S1P5 agonist Etrasimod, has been shown to be an effective treatment option in ulcerative colitis patients [32]. Whether these receptors are affected by CerS5 in T-cells has to be proven by further experiments. Helke et al. did not investigate the T-cell status in CerS6-ko mice after DSS treatment, and thus the fact that T-cells contribute to severe colitis in these mice cannot be excluded [9]. Intraepithelial T-cells (IET) that are located in the epithelium and have different protective functions in the colon. After activation with IL-2, TNF and interferon- γ, especially CD^8+^ IETs, rapidly release cytotoxic substances such as granzyme B and perforin, and thereby very efficiently kill invading bacteria. However, it has also been shown that IETs are important for maintaining the integrity of the intestinal mucosa by maintaining epithelial cell proliferation [19,33]. Because IETs were already reduced in CerS5-ko control mice, we hypothesize that the lack of the protective effect of IETs is in part responsible for the higher susceptibility of CerS5-ko mice to DSS-induced inflammation and CAC. 

Our data indicate that a global knockdown of CerS5 influences T-cell migration/activation, leading to an imbalance of cytokines in the colon of control mice. However, further studies are needed to clarify how CerS5 depletion impairs T-cell signaling and how this interferes with acute inflammation and CAC. 

## 4. Materials and Methods 

### 4.1. Cell and Reagents

Jurkat cells (human T-cells; ATCC TIB-152) were cultured in RPMI-1640 medium supplemented with 2 mM glutamate and 10% fetal calf serum (FCS) at 37 °C in an atmosphere containing 5% CO_2_. The human colon cancer cell lines Caco-2 (ATCC HTB-37) and HCT-15 (ATCC CCL-225) were cultivated in MEM Medium (+ Earle’s Salts and L-glutamine) supplemented with 10% FCS and 1% non-essential amino acids (Sigma M7145) (for Caco-2), or in RPMI-1640, supplemented with 10% FCS and 1% Glutamax (for HCT-15) at 37 °C in an atmosphere containing 5% CO_2_.

### 4.2. Generation of CerS5 Knockdown Cells

CerS5 was downregulated in Caco-2, HCT-15, and Jurkat cells by viral transduction of CerS5-specific shRNA (GIPZ lentiviral vector system, Thermo Scientific), as descripted previously [34]. As a negative control (NC), we used a scrambled shRNA (GE Dharmacon, Lafayette, CO, USA). In brief, CerS5 shRNA or NC shRNA containing vectors together with lentiviral packaging vectors were transfected into HEK293T cells using calcium phosphate. After 48 h, virus particles were harvested with the PEG-it VPS Kit (SBI System Biosciences, CA, USA) and used for transfection of Caco-2, HCT-15, and Jurkat cells. Stable cell lines were selected with puromycin (InvivoGen, Toulouse, France; 1 μg/mL for Caco-2 and Jurkat, or 3 μg/mL for HCT-15). GFP expression in transduced cells was used as transduction efficacy control and was monitored by fluorescence microscopy.

### 4.3. Animal Models

To analyze the role of CerS5 in inflammation and colon cancer development, we used CerS5 knockout (ko) mice and CerS5 fl/fl mice (obtained from Prof. Martin Krönke and Prof. Jens Brüning (Institute for Medical Microbiology, Immunology and Hygiene (IMMIH); University of Cologne, Cologne, Germany)) crossed with B6.Cg-Tg(Vil-cre) mice (The Jackson Laboratory (by Charles River, Sulzfeld, Germany)) in the dextran sodium sulfate (DSS)-induced acute colitis and the azoxymethane (AOM)/DSS colitis-associated colon cancer (CAC) model. The generation of CerS5-ko mice is based on the deletion of exon 4 of the CerS5 gene by the insertion of exon 4 flanking loxP sites and crossing these mice with Cre-deleter mice, as described in [35]. The deletion of exon 4 results in a frameshift in downstream exons that prevents the translation of the catalytic LAG1 domain [35]. Mice with a specific deletion of CerS5 in villus and crypt epithelial cells of the small and large intestine were generated by interbreeding CerS5fl/fl mice with mice expressing the Cre-recombinase under the control of the villin 1-promoter (Vil1Cre). Because no specific anti-CerS5 antibody is available, we provided the immunohistochemical staining of Cre in colon epithelial cells in CerS5fl/fl mice and a PCR experiment testing for deletion of exon 4 in CerS5fl/fl mice using colon and liver tissue according to [36] (Figure S3). CerS5 delta PCR Primer:

CerS5FWD: 5′CAACATGATTCCAGTCTGTTCC3’
CerS5Int4_6: 5′ GGCACGAAGAAAGTCTGGAG3’
CerS5floxREV: 5′CTCACTATGTAACCATGCTG3’

The animal experiments followed ethics guidelines and were approved by the local ethic committee for animal research (V54-19c20/15-FK 10/13, regional council, Darmstadt, Germany). The acute DSS and the AOM/DSS-CAC mice model were performed as described in our previous paper [11]. In brief, for the acute inflammation model, 2% (w/v) DSS (MP Biomedicals) was applied to drinking water for 6 days and was exchanged afterwards. For the chronic inflammation model, 10mg/kg azoxymethane (Sigma-Aldrich) was initially injected intraperitoneally at day 0, followed by 2% DSS application in drinking water for 6 days. To induce a chronic inflammation, we repeated DSS treatment twice after a recovery of 14 days between each DSS cycle. To determine disease progression, we monitored body weight, stool consistency, and bleeding over time and we used this to estimate a score. The data are blotted against time, and area under the curve was calculated with the trapezoidal rule:

(1) $$AUC_{\left(ti-ti-1\right)}=\frac{f\left(t_{i}\right)+f\left(t_{i-1}\right)}{2}\times \left(ti-t_{i-1}\right)$$

(2) $$AUC_{\left(b-a\right)}=\sum_{t=a+1}^{b}AUC_{\left(ti-ti-1\right)}$$

At the end of each experiment (acute inflammation model 10 days, CAC model 10–12 weeks), the mice were killed and blood and tissues were collected.

### 4.4. In Vivo Imaging of Colon Inflammation

Bioluminescence imaging was performed with an IVIS Lumina Spectrum that employs XENOGEN technology (Caliper LifeSciences) under 1.5–2% isoflurane anesthesia. Images of three mice per group were captured and analyzed with Living Image software (Perkin Elmer). The inflammation of the gastrointestinal tract was assessed with the bioluminescent XenoLight RediJect Inflammation Probe (Perkin Elmer), which allows in vivo assessment of myeloperoxidase (MPO) activity. The abdominal region was shaved to reduce the absorption of light. Inflammation Probe (200 mg/kg, around 150 μL) was injected intraperitoneally 4 to 10 min before imaging. The IVIS settings were Epi-BLI, Em filter open, Ex filter block, fstop 1, binning 8, exposure 120 s, and focus B 6.5 cm.

### 4.5. Tissue Preparation for Molecular and Histological Studies 

Colons from mice were cleaned by flushing with ice cold Dulbecco’s phosphate-buffered saline (DPBS) without Ca_2_^+^ and Mg_2_^+^ (1×, Gibco by Life Technologies, Thermo Fisher Scientific) with a syringe through the colon. Afterwards, the colon was cut longitudinally. To evaluate colon length and tumor volume, we used FIJI to take and analyze pictures [37]. Small pieces (5–10 mg) were frozen quickly on dry ice for LC–MS/MS and RNA isolation. In terms of the residual colon, it either was cut further in 0.5 cm pieces for flow cytometric analysis or used for immunohistochemistry. For flow cytometry, spleen and blood samples were also taken. 

### 4.6. Preparation for FACS

To characterize the extent of inflammation induced by DSS, we detected immune cell surface markers in single cell suspensions from different tissues (blood, spleen, and colon) by flow cytometry. For single cell preparation of the white blood cells (WBC), we supplemented EDTA blood with the same volume of HEPES. Subsequently, erythrocytes were lysed and the remaining single cell suspension was resuspended in FACS Flow (BD Bioscience). Spleens were prepared by disrupting the tissue mechanically and running it through a 70 μm Corning cell strainer. The cells were subjected to RBC lysis once, were washed in PBS, and were resuspended in FACS Flow. To detect the immune cells infiltrating the colon, we generated single cell suspensions of lamina propria cells (LPs) and intraepithelial lymphocytes (IELs) from the colon using the lamina propria dissociation kit (Miltenyi Biotec) according to the manufacturer’s instructions (described previously in [11]). 

After preparing single cell suspensions, cells were blocked with FcR Blocking Reagent (Milteneyi Biotec, Bergisch Gladbach, Germany) for 15 min, followed by a 15 min staining with an antibody cocktail (CD3-PE-CF594, CD4-BV711, CD11b-BV510, CD11c-AlexaFlour700, CD14-PE, CD19-APC-H7, CD25-PE-Cy7, CD80-FITC, GITR-FITC, Ly6G-APC-Cy7, MHC-II-BV605, NK1.1-PE, 7-AAD-PE-Cy5 (BD, Heidelberg, Germany), CD45-Vioblue (Miltenyi Biotec, Bergisch Gladbach, Germany), CD8-eFluor655, CD36-APC, Ly6C-PerCP-Cy5.5 (eBioscience, Frankfurt, Germany), and F4/80-PE-Cy7 (BioLegend, Fell, Germany)) at room temperature. Afterwards, single cell suspensions were washed and resuspended in FACS Flow for flow cytometry. Samples were acquired with a LSRII/ Fortessa flow cytometer (BD Bioscience, Heidelberg, Germany) and were analyzed using FlowJo software v10 (Treestar, Ashland, MA, USA).

### 4.7. Eosin–Hematoxylin Staining

For immunohistochemistry of the colon, the longitudinally opened colon was rolled from distal to proximal, embedded with Tissue-Tek O.C.T. Compound (Sakura Finetek) in Tissue-Tek Intermediate Cryomold (Sakura Finetek), frozen quickly on dry ice, and subsequently stored at −80 °C. Afterwards, the colon role was cut into 10 μm tissue sections and then was stained with eosin and hematoxylin. Finally, slides were dehydrated using an ascending alcohol series and xylene and mounted with pertex (Medite). The images were taken with the Keyence BZ-9000 microscope.

### 4.8. Immunohistochemistry

For immunohistochemistry tissues, slides were fixated in 4.5% PFA. After fixation and washing twice with PBS, slices were permeabilized in PBS containing 0.025% Triton X-100 PBST (PBST) for 13 min. After blocking the slices in PBS containing 3% bovine serum albumin (BSA) for 90 min at RT, the first primary antibody (Cre (NB100-56133; Novus Biologicals) 1:150, ZO-1 1:200, occludin (OC-3F10, Invitrogen) 1:300 diluted in PBS containing 1% BSA and 1% NGS) was incubated overnight. For fluorescence labeling, secondary Alexa Fluor 647-anti-rabbit antibody or Cy3-anti-mouse antibody were diluted 1:1000 in the same buffer as the primary antibody and incubated at RT for 2 h. 4′,6-Diamidine-2′-phenylindole dihydrochloride (DAPI) was diluted 1:1000 in PBS and stained for 10 min. After washing with PBS, the slices were mounted with PolyAquamount (Polyscience). Pictures were taken with a Zeiss Axio Imager Z1 microscope with Apoptome unit.

### 4.9. RNA Isolation and qRT-PCR

To analyze the expression levels of CerS in human cell lines, we isolated mRNA from Jurkat, Caco-2, and HCT-15 cells using the Qiagen RNA isolation kit following the manufacturer’s instructions. The RNA was eluted in 30 μL DNase/RNase-free water (Qiagen GmbH, Hilden, Germany). To analyze cytokine mRNA expression within the colon, we used 5 mg of colon tissue for RNA isolation using an RNAqueous-Micro kit (invitrogen). The isolation was performed according the to the manufactures protocol. The RNA was eluted in 20 μL according the instructions. For cDNA synthesis, 800 ng RNA was reverse-transcribed to cDNA using the Verso cDNA Synthesis kit (Thermo Fisher Scientific, Waltham, MA, USA). We applied 1 μL of cDNA to detect mRNA expression levels of cytokines or of CerS1, CerS2, CerS3, CerS4, CerS5, CerS6, and RPL37A using EvaGreen (Bio-Sell) with an ABI Prism 7500 Sequence Detection System (Applied Biosystems, Foster City, CA, USA) or SYBR Select (Applied Biosystems, Foster City, CA, USA) with the QuantStudio 5 system (Applied Biosystems, Foster City, CA, USA). Relative mRNA expression was determined using the comparative CT (cycle threshold) method. CerS expression level was normalized to the expression level of human 60 S ribosomal protein L37 (RPL37A), whereas cytokine expression level was normalized to the murine GAPDH. For the primer list, see Table 1.

### 4.10. LC–MS/MS

Sphingolipid concentrations of tissues and cells were quantified by high-performance liquid chromatography–tandem mass spectrometry (LC–MS/MS). The tissue samples were first mixed with water and homogenized to a suspension of 0.05 mg/μL tissue using a swing mill (Retsch, Haan, Germany) with four zirconium oxide grinding balls for each sample (25 Hz for 2.5 min). A total of 40 μL of the tissue suspension (in total 2 mg tissue) was mixed with 160 μL water, 200 μL extraction buffer (citric acid 30 mM, and disodium hydrogen phosphate 40 mM), and 20 μL of the internal standard solution containing 10C16:0 Cer-d31, C18:0 Cer-d3, C17:0 LacCer, C18:0 DHC-d3, C16:0 LacCer-d3, C18:0 GluCer-d5 (all avanti polar lipids, Alabaster, USA), and C24:0 Cer-d4 (Chiroblock GmbH, Bitterfeld-Wolfen, Germany; 400 ng/mL each). The mixture was extracted twice with 600 μL methanol/chloroform/hydrochloric acid (15:83:2, v/v/v). The collected lower organic phases were evaporated at 45 °C under a gentle stream of nitrogen and reconstituted in 100 μL of tetrahydrofurane/water (9:1, v/v) containing 0.2% formic acid and 10 mM ammonium formate. For colon cell lines, the volumes of the protocol were changed. Therein, cell pellets were suspended with 150 μL water, 150 μL extraction buffer, and 20 μL internal standard. The mixture was extracted once with 1000 μL methanol/chloroform/hydrochloric acid (15:83:2, v/v/v), and after evaporation was reconstituted in 200 μL of tetrahydrofurane/water (9:1, v/v) containing 0.2% formic acid and 10 mM ammonium formate. After sample preparation, both tissue and cell extracts were measured using the same method. Amounts of sphingolipids were analyzed by liquid chromatography coupled to tandem mass spectrometry (LC–MS/MS). An Agilent 1200 series binary pump (Agilent Technologies, Waldbronn, Germany) equipped with a Zorbax EclipsePlus C18 column (50 mm × 2.1 mm ID, 1.8 μm particle size; Agilent Technologies, Waldbronn, Germany) was used for chromatographic separation. The column temperature was set at 55 °C. The HPLC mobile phases consisted of water with 0.2% formic acid and 2 mM ammonium formate (A) and acetonitrile/isopropanol/acetone (50:30:20, v/v/v) with 0.2% formic acid (B). For separation, a gradient program was used. The initial buffer composition 55% (A)/45% (B) was held for 0.6 min. Then, within 3.9 min linearly changed to 0% (A)/100% (B) and held for 8.5 min. Subsequently, the composition was linearly changed within 1.0 min to 55% (A)/45% (B), and then the column was re-equilibrated for 5.5 min. The total running time was 19.5 min. The flow rate was 0.4 mL/min from start until 6.0 min; within 0.5 min it was decreased to 0.3 mL/min and held for 3 min. After this, it was slowly increased again within 4 min. The injection volume was 10 μL. To improve ionization, acetonitrile with 0.1% formic acid was infused post-column using an isocratic pump at a flow rate of 0.15 mL/min. The MS/MS analyses were performed using a mass spectrometer 5500QTRAP (Sciex, Darmstadt, Germany), operating as triple quadrupole, equipped with a Turbo V Ion Source operating in positive electrospray ionization mode. The MS parameters were set as follows: Ionspray voltage, 5500 V; ion source temperature, 450 °C; curtain gas, 37.5 psi; collision gas, 9 psi; nebulizer gas, 45 psi; and heating gas, 65 psi. The analysis was carried out in multiple reaction monitoring (MRM) mode. Data acquisition was performed using Analyst Software V 1.6 and quantification was performed with MultiQuant Software V 3.0 (both Sciex, Darmstadt, Germany), employing the internal standard method (isotope dilution mass spectrometry). Calibration curves were calculated by linear regression with 1/× or 1/×2 weighting. Variations in accuracy of the calibration standards were less than 15% over the whole range of calibration, except for the lower limit of quantification, where a variation in accuracy of 20% was accepted. 

### 4.11. TEER Measurements

To assess the barrier functionality of epithelial colon cancer cells in vitro, we determined transepithelial electrical resistance (TEER) using cellZscope (nanoAnalytics), which ensures a measurement of barrier function under physiological conditions. We seeded 5 × 10^4^ HCT-15 cells in 1 μm transparent 24-well ThinCert Inserts (Greiner Bio-One), which were previously labelled with poly-L-lysine. Caco-2 cells (3 × 10^4^ per insert) were seeded on BSA-coated inserts. Every 3 days, half of the medium was exchanged with fresh medium. After 4 days, the complete medium was exchanged with fresh medium. CellZscope device was set to stimulate the cells with 1 Hz to 100 kHz and the impedance was measured each hour. The calculation of the capacity and the electrical resistance was performed by the CellZscope software. 

### 4.12. Permeability Assay

The permeability of colon cancer cells in vitro was analyzed using fluorescein isothiocyanate (FITC)-dextran. We added 100 μg/mL FITC (average molecular weight: 40,000 by Sigma Aldrich) to the upper insert when cells reached a plateau and the impedance was on a saturated and constant level. Then, 20 μL from the apical medium and 66.7 μL from the lateral medium were taken at indicated time points up to 48 h and transferred to a black polysterol 384-well plate (Greiner Bio-One). At the end of the experiment, fluorescence was measured using the EnSpire Multimode Plate Reader (perkinElmer) at an excitation wavelength of 496 nm and an emission wavelength of 530 nm, with 100 flashes. To calculate the FITC content from the emission, we included a standard curve. 

### 4.13. Detection of Proteins by Western Blot 

Jurkat cells were stimulated for 0–48 h with 200 units/mL IL-2 (PeproTech GmbH, Hamburg, Germany) and CD2/3/28 activation beads (Miltenyi Biotec, Bergisch Gladbach, Germany) in a 1:1 beads-to-cell ratio. Thereafter, cells were centrifuged, lysed in PhosphoSafe Extraction Reagent (Merck, Darmstadt, Germany), and sonicated. The lysate was centrifuged, and the supernatants were collected and stored at −80 °C. Protein concentration was determined using the Bradford method [38]. Equal amounts of protein (15–50 μg) were separated by 12% sodium dodecylsulfate polyacrylamide gel electrophoresis (SDS-PAGE) and then electroblotted on nitrocellulose membranes (Amersham Life Science, Freiburg, Germany). Membranes were blocked with 5% milk powder in 0.1% Tween 20 in PBS and immunostained for 48 h at 4 °C with the respective primary antibody directed against *p*-p65 (phospho-NF-κB p65 (Ser536), 93H1 rabbit mAb; 1:200) and p65 (NF-κB p65 L8F6 mouse mAb; 1:200) (Cell Signaling, Leiden, Netherlands), and for 30 min with β-actin (AC-15 #A5441, mouse mAb or #A2066 rabbit pAb; 1:1000) (Sigma-Aldrich, St. Louis, MO, USA) diluted in 5% milk powder in 0.1% Tween 20 in PBS. Membranes were washed twice with 0.1% Tween 20 in PBS, followed by incubation with an IRDye800- or IRDye700-conjugated secondary antibody (BIOTREND Chemikalien GmbH, Köln, Germany) in 5% milk powder in 0.1% Tween 20 in PBS (1:10,000) for 35 min at RT. Membranes were analyzed on the Odyssey infrared scanner (LI-COR Biosciences, Bad Homburg, Germany). Quantitative densitometric analysis was performed using the Image Studio Lite software (LI-COR Biosciences, Bad Homburg, Germany).

### 4.14. Statistics

Sphingolipid levels, mRNA levels, and flow cytometry data are presented as mean ± SEM (standard error of the mean). Statistical analyses were performed with GraphPad Prism 7 software. Significant differences between groups were assessed using one-way ANOVA for three or more groups or two-tailed, two-sided Student’s *t*-tests for two groups. In case of significant ANOVAs, groups were mutually compared with *t*-tests employing a correction of alpha according to Tukey. For time courses, for example of body weight, and colitis scores, we calculated the area under the curve (AUC) values using GraphPad Prism 7 and analyzed them via Student’s *t*-tests.

## 5. Conclusions

Our data show that CerS5-ko mice were more susceptible to DSS-induced colitis and AOM/DSS-induced CAC. A reduced number of T-cells that was already the case in untreated CerS5-ko mice in different tissues and the epithelium of the colon might have been responsible for this effect. Furthermore, TCR signaling was found to be impaired in CerS5-downregulated human T-cells, which also had an influence on the expression of other CerS in these cells. Due to the high analogy of our data in CerS5-ko mice and data of CerS6-ko mice, it appears that CerS5 and CerS6 similarly influenced T-cells. Further studies are needed to investigate the role of CerS in T-cell function.

## Figures and Tables

**Table 1:** qRT-PCR primer used for mRNA analysis. The list contains both human (h) and murine (m) primers. Human CerS iosenzymes are termed as “Lass” (longevity assurance homologue of yeast lag1).

Primer	Forward 5′–3′	Reverse 5′–3′
RLP37A	ATT GAA ATCA GCC AGC ACG C	AGG AAC CAC AGT GCC AGA TCC
RLP13A (Jurkat)	CTCAAGGTGTTTGACGGCATCC	TACTTCCAGCCAACCTCGTGAG
hLass1	CCT CCA GCC CAG AGA T	AGA AGG GGT AGT CGG TG
hLass2	CCA GGT AGA GCG TTG GTT	CCA GGG TTT ATC CAC AAT GAC
hLass3	CCT GGC TGCTAT TAG TCT GAT	TCA CGA GGG TCC CAC T
hLass4	CTG GTG GTA CCT CTT GGA GC	CGT CGC ACA CTT GCT GAT AC
hLass5	CAA GTA TCA GCG GCT CTG T	ATT ATC TCC CAA CTC TCA AAG A
hLass6	AAG CAA CTG GAC TGG GAT GTT	AAT CTG ACT CCG TAG GTA AAT ACA
mIL-4	GTC ATC CTG CTC TTC TTT CTC G	CTG TGG TGT TCT TCG TTG CTG
mIL-1ß	TCC AGG ATG AGG ACA TGA G	GAG CCT GTA GTG CAG TTG
mIL-6	TAG TCC TTC CTA CCC CAA TTT CC	TTG GTC CTT AGC CAC TCC TTC
mIL-10	TGC CAA GCC TTA TCG GAA ATG	ACT CTT CAC CTG CTC CAC TGCC
mIL-17	TCT GTG TCT CTG ATG CTG TTG CTG	CAG GGT CTT CAT TGC GGT GG
mINF-y	CAC GGC ACA GTC ATT GAA AGC	CAC CAT CCT TTT GCC AGT TCC
mGAPDH (MP205604, ORIGENE)	CAT CAC TGC CAC CCA GAA GAC TG	ATG CCA GTG AGC TTC CCG TTC AG

## Supplementary Materials

The following are available online at https://www.mdpi.com/2072-6694/12/7/1753/s1, Figure S1: IHC of colon roles. Exemplary pictures of whole colons from CerS5-wt and CerS5-ko mice and immunohistological pictures of colon roles (10 µm tissue sections stained with eosin and hematoxylin) with tumor regions (red boxes). Images were taken with the Keyence BZ-9000 microscope with 10-fold magnification and recomposed by the software, Figure S2: Downregulation of CerS5 in Colon cancer cell lines. Downregulation of CerS5 expression by lentiviral transduction of Caco-2 and HCT-15 cells with CerS5-targeting shRNA. In both human colon cancer cell lines, CerS5 mRNA expression could be downregulated, whereas the expression levels of other CerS were not affected. Downregulation of CerS5 was accompanied by significant decreases in the long-chain ceramides C14:0-Cer and C16:0-Cer in Caco-2 cells. Data are mean +/− SEM of two (HCT-15) or three (Caco-2) independent experiments, Figure S3: (**A**) PCR experiments testing for detection of exon 4 specific for *CerS5* in colon and liver of control and CerS5fl/fl VilCre mice. CerS5-floxed-bands appear at 328 bp and CerS5△ (deletion)-bands at 567 bp and CerS5wt at 162 bp. Mouse no.24 and 27 Lass5wt/Vil Cre negative; 25 and 28 Lass5wt/Vil Cre positive. (**B**) Immunohohistochemical staining of Cre in CerS5fl/fl Vil Cre positive and negative mice. Cre is localized in the nucleus of colon epithelial cells, Figure S4: The whole western blot image.

## Abbreviations

azoxymethane (AOM); ceramide (Cer); ceramide synthase (CerS); colitis-associated colon cancer (CAC); control (ctrl), 4′,6-diamidine-2′-phenylindole dihydrochloride (DAPI); dextran sodium sulfate (DSS); dihydro-ceramide (dhCer); glycosyl-ceramide (GluCer); sodium butyrate (NaBt); sphinganine-1 phosphate (SA1P), sphingosine-1 phosphate (S1P); transepithelial electrical resistance (TEER); ulcerative colitis (UC).
