# Supplementary material for: Ceramide Synthase 5 Deficiency Aggravates Dextran Sodium Sulfate-Induced Colitis and Colon Carcinogenesis and Impairs T-Cell Activation
Source: Cancers (Basel). 2020 Jul 1;12(7):1753. doi: 10.3390/cancers12071753 (PMC7409364; doi:10.3390/cancers12071753)
Supplement: Supplementary file 1 [file cancers-12-01753-s001.pdf]

cancers

Article

Ceramide Synthase 5 Deficiency Aggravates Dextran Sodium Sulfate-Induced Colitis and Colon Carcinogenesis and Impairs T-Cell Activation

Khadija El-Hindi [1,†], Sebastian Brachtendorf [1,†], Jennifer Christina Hartel [1,5], Stephanie Oertel [1], Kerstin Birod [1], Sandra Trautmann [1], Dominique Thomas [1], Thomas Ulshöfer [2], Andreas Weigert [3], Olaf Utermöhlen [4], Martin Krönke [4] and Sabine Grösch [1,*]

1 Institute of Clinical Pharmacology, Faculty of Medicine, Goethe-University Frankfurt; Frankfurt 60590, Germany; El-Hindi@med.uni-frankfurt.de (K.E.-H.); Sebastian.Brachtendorf@web.de (S.B.); jhartel@med.uni-frankfurt.de (J.C.H.); stephi1106@hotmail.com (S.O.); k.birod@med.uni-frankfurt.de (K.B.); trautmann@med.uni-frankfurt.de (S.T.); thomas@med.uni-frankfurt.de (D.T.)

2 Fraunhofer Institute for Molecular Biology and Applied Ecology IME, Project Group Translational Medicine and Pharmacology (TMP), Frankfurt 60596, Germany; Thomas.Ulshoefer@ime.fraunhofer.de

3 Institute of Biochemistry I-Pathobiochemistry, Faculty of Medicine, Goethe-University Frankfurt; Frankfurt, Germany; weigert@biochem.uni-frankfurt.de

4 Center for Molecular Medicine Cologne, Institute for Medical Microbiology, Immunology and Hygiene, University of Cologne, Cologne 50935, Germany; olaf.utermoehlen@uk-koeln.de (O.U.); m.kroenke@uni-koeln.de (M.K.)

5 Department of Life Sciences, Goethe-University Frankfurt, Frankfurt, Germany

* Correspondence: groesch@em.uni-frankfurt.de; Tel.: +49/69-6301-7820; Fax: +49/69-6301-7660

† Authors contributed equally.

Supplementary

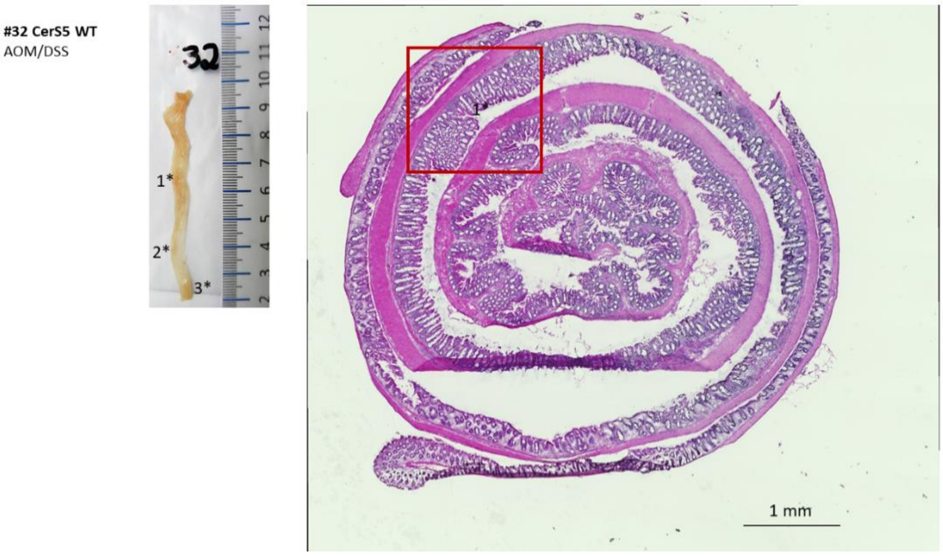

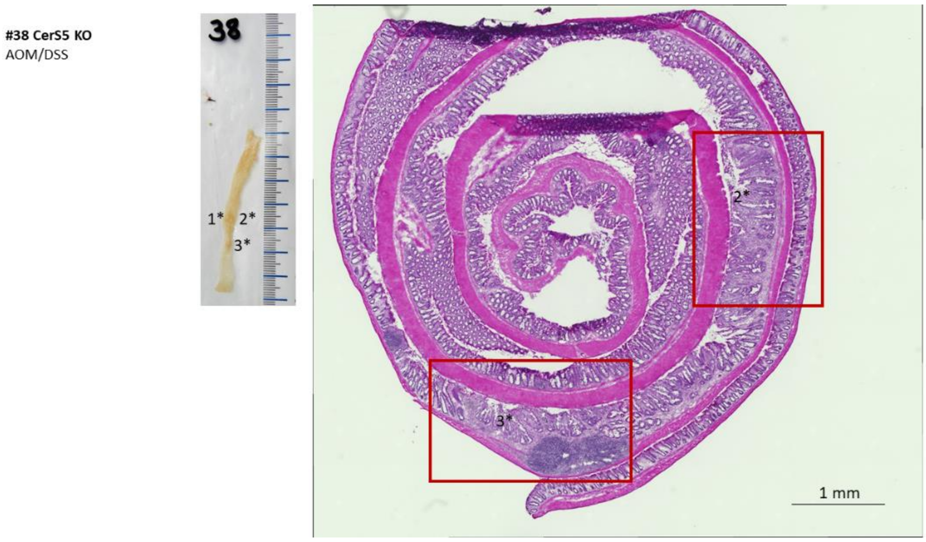

Figure S1. IHC of colon roles. Exemplary pictures of whole colons from CerS5-wt and CerS5-ko mice and immunohistological pictures of colon roles (10 µm tissue sections stained with eosin and hematoxylin) with tumor regions (red boxes). Images were taken with the Keyence BZ-9000 microscope with 10-fold magnification and recomposed by the software.

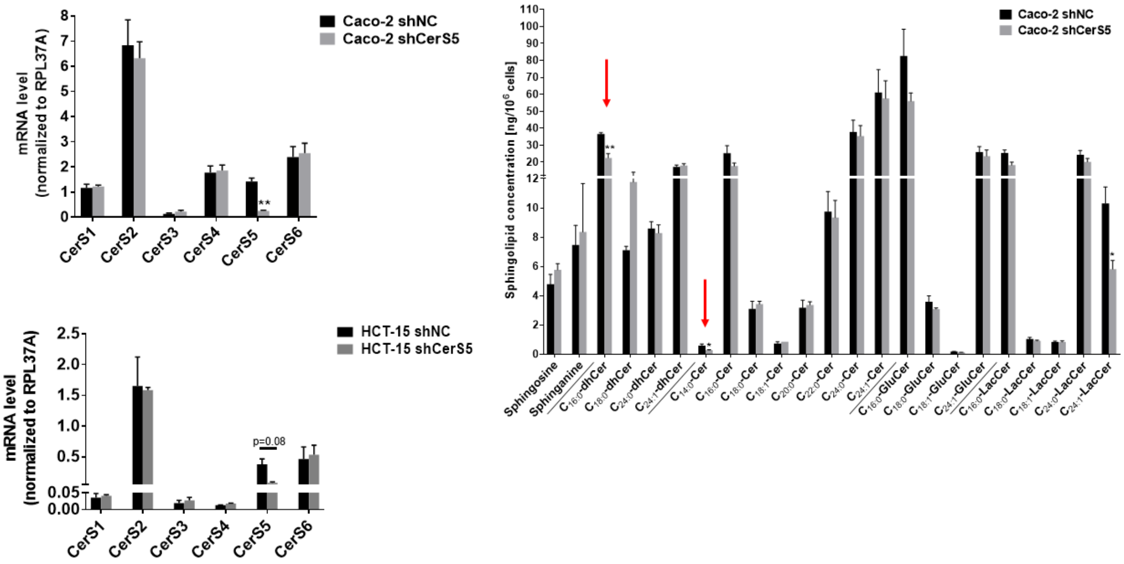

Figure S2. Downregulation of CerS5 in Colon cancer cell lines. Downregulation of CerS5 expression by lentiviral transduction of Caco-2 and HCT-15 cells with CerS5-targeting shRNA. In both human colon cancer cell lines, CerS5 mRNA expression could be downregulated, whereas the expression levels of other CerS were not affected. Downregulation of CerS5 was accompanied by significant decreases in the long-chain ceramides C14:0-Cer and C16:0-Cer in Caco-2 cells. Data are mean +/- SEM of two (HCT-15) or three (Caco-2) independent experiments.

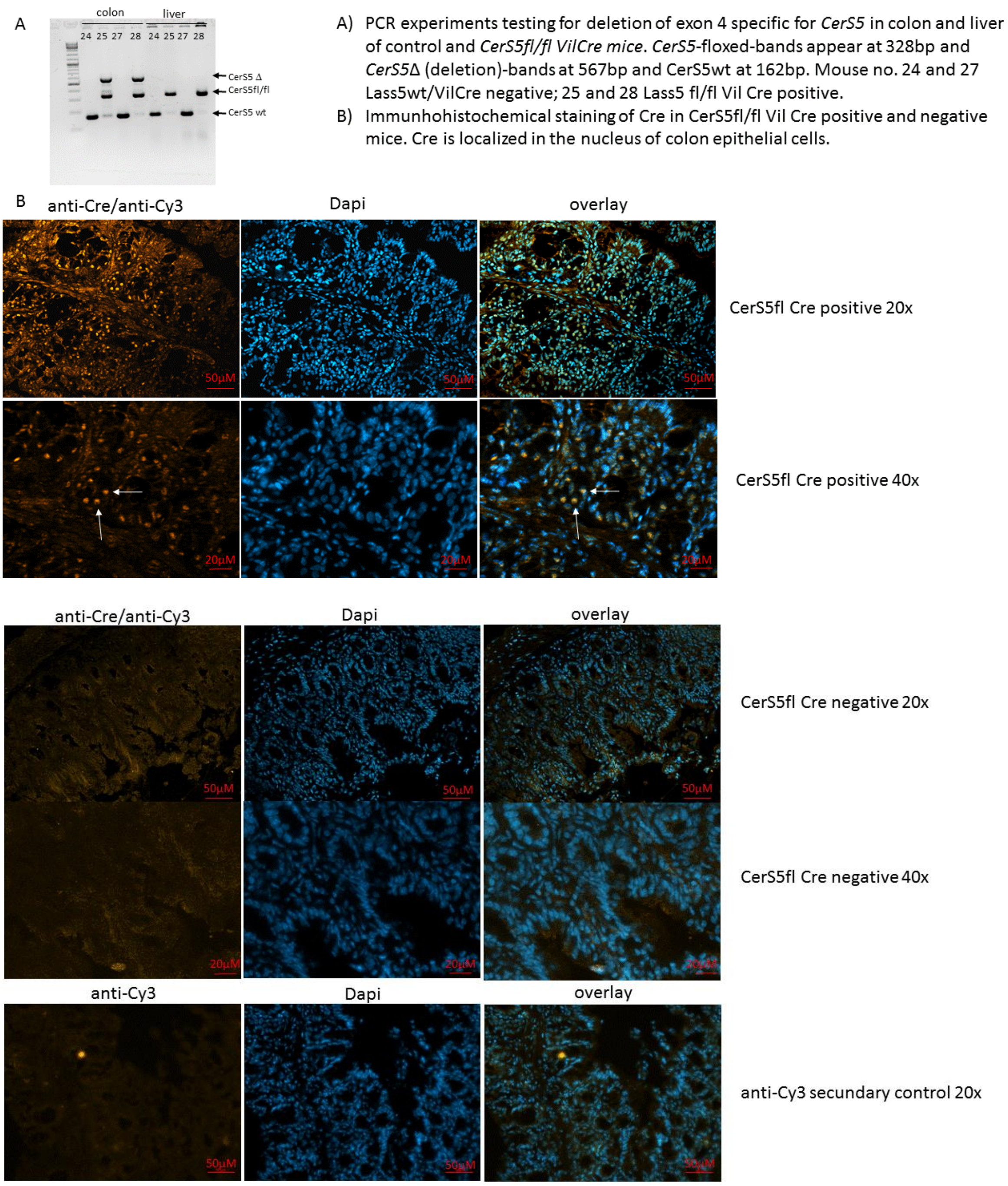

Figure S3. (**A**) PCR experiments testing for detection of exon 4 specific for *CerS5* in colon and liver of control and CerS5fl/fl VilCre mice. CerS5-floxed-bands appear at 328 bp and CerS5△ (deletion)-bands at 567 bp and CerS5wt at 162 bp. Mouse no.24 and 27 Lass5wt/Vil Cre negative; 25 and 28 Lass5wt/Vil Cre positive. (**B**) Immunohohistochemical staining of Cre in CerS5fl/fl Vil Cre positive and negative mice. Cre is localized in the nucleus of colon epithelial cells.

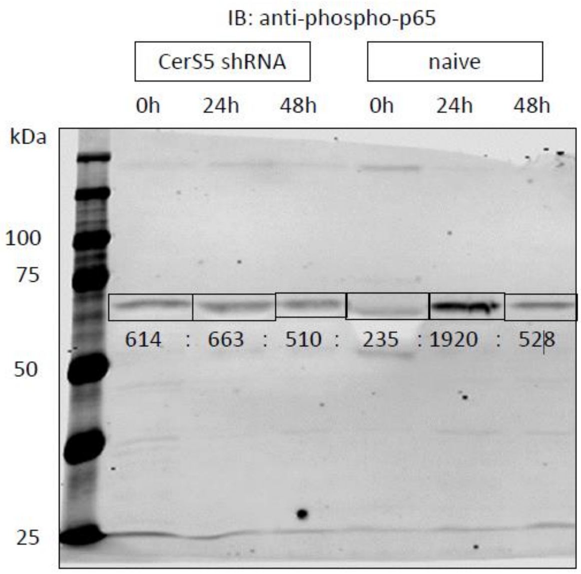
IB: anti-phospho-p65
CerS5 shRNA
naive
0h 24h 48h 0h 24h 48h
kDa
100
75
50
25
614 : 663 : 510 : 235 : 1920 : 528

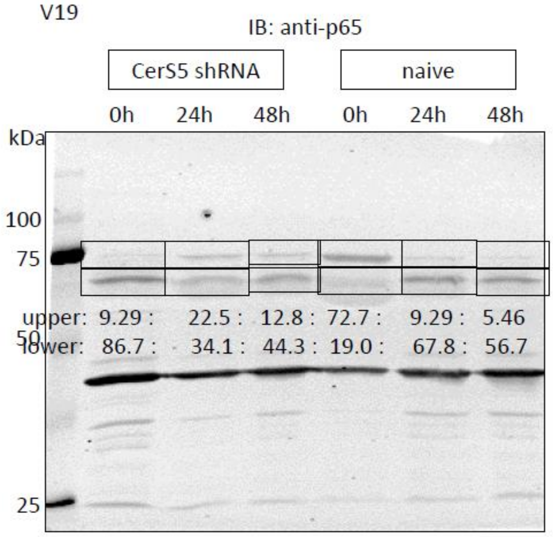
V19
IB: anti-p65
CerS5 shRNA
naive
0h 24h 48h 0h 24h 48h
kDa
100
75
50
25
upper: 9.29 : 22.5 : 12.8 : 72.7 : 9.29 : 5.46
lower: 86.7 : 34.1 : 44.3 : 19.0 : 67.8 : 56.7

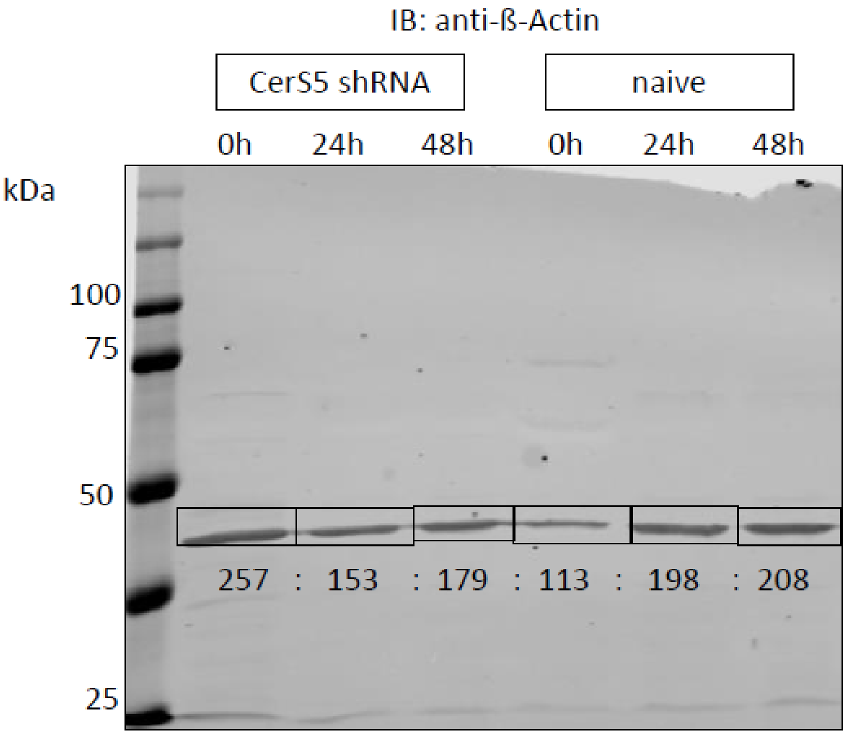
IB: anti-ß-Actin
CerS5 shRNA
naive
0h 24h 48h 0h 24h 48h
kDa
100
75
50
25
257 : 153 : 179 : 113 : 198 : 208

V20 (Figure 6)

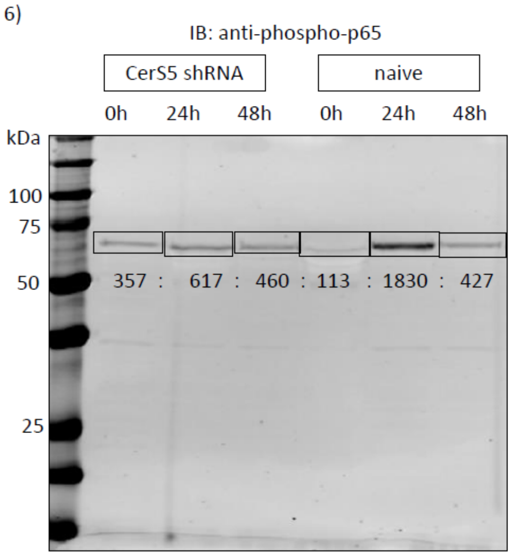

V20

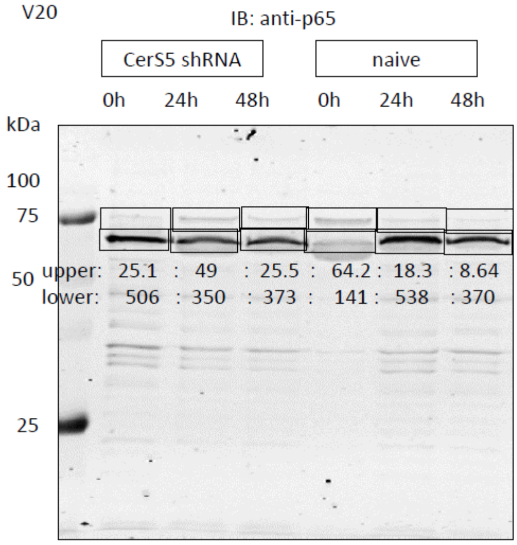

V20 (Figure 6)

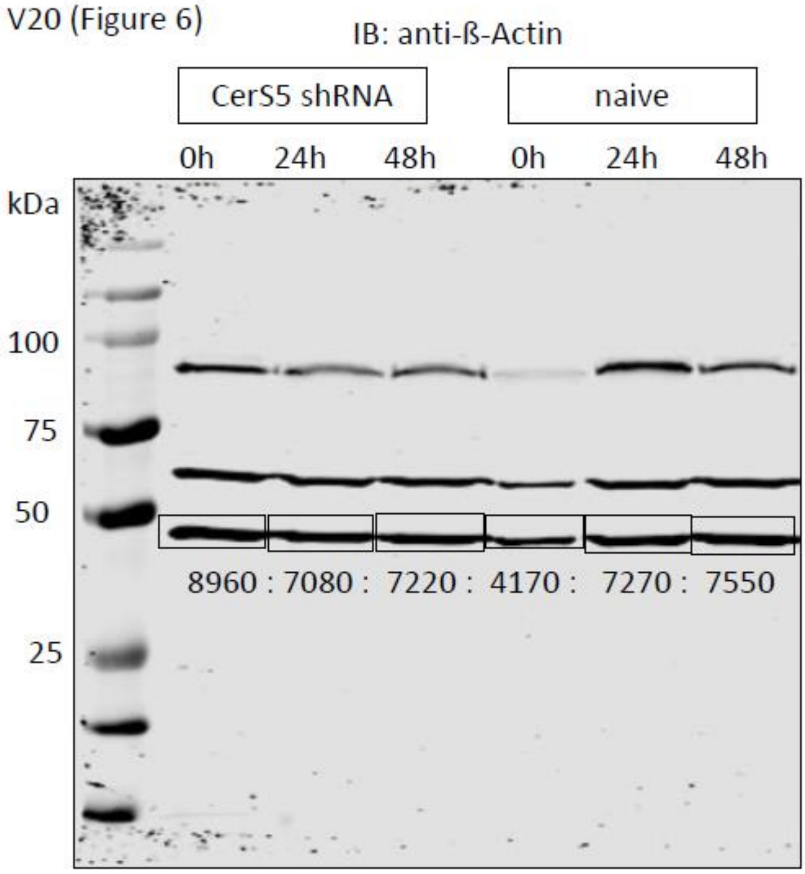

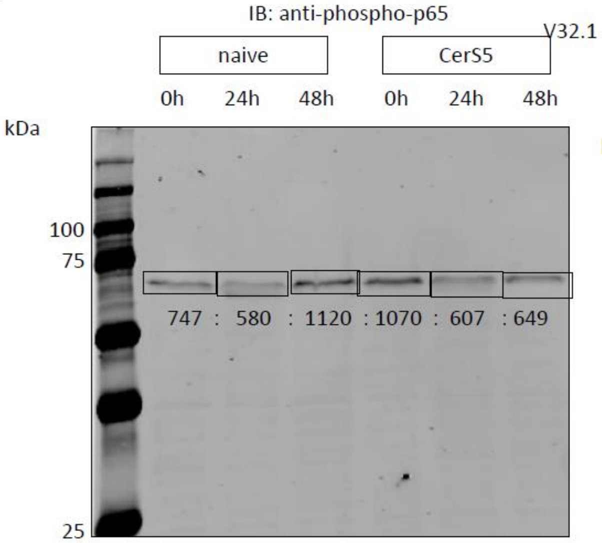
IB: anti-phospho-p65
V32.1
naive
CerS5
0h 24h 48h 0h 24h 48h
kDa
100
75
25
747 : 580 : 1120 : 1070 : 607 : 649

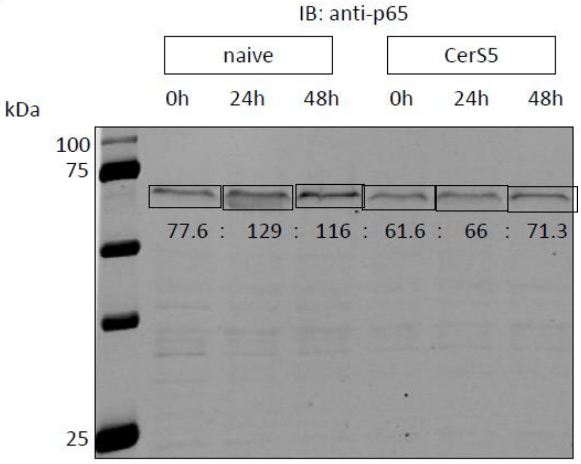
IB: anti-p65
naive
CerS5
0h 24h 48h 0h 24h 48h
kDa
100
75
25
77.6 : 129 : 116 : 61.6 : 66 : 71.3

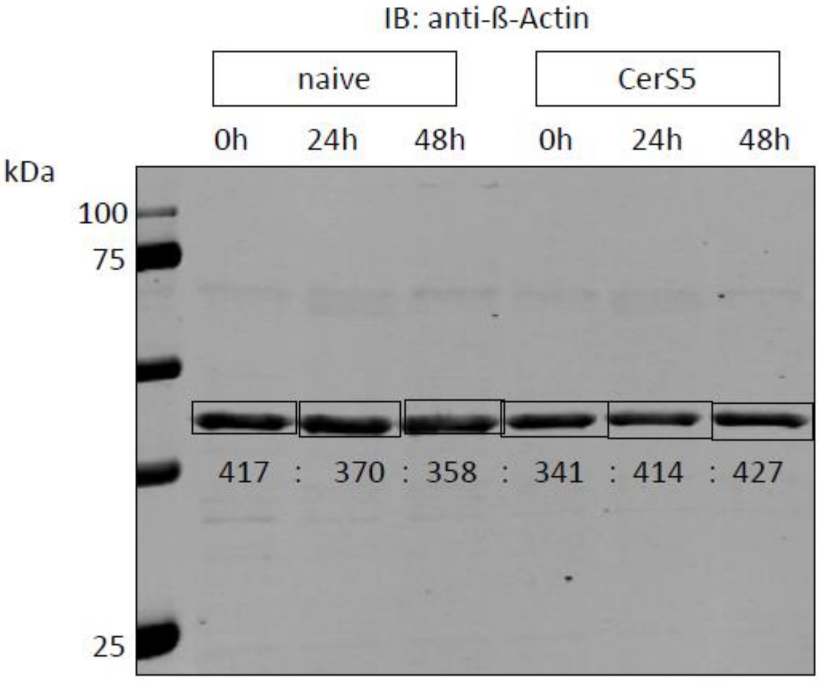
IB: anti-ß-Actin
naive
CerS5
0h 24h 48h 0h 24h 48h
kDa
100
75
25
417 : 370 : 358 : 341 : 414 : 427

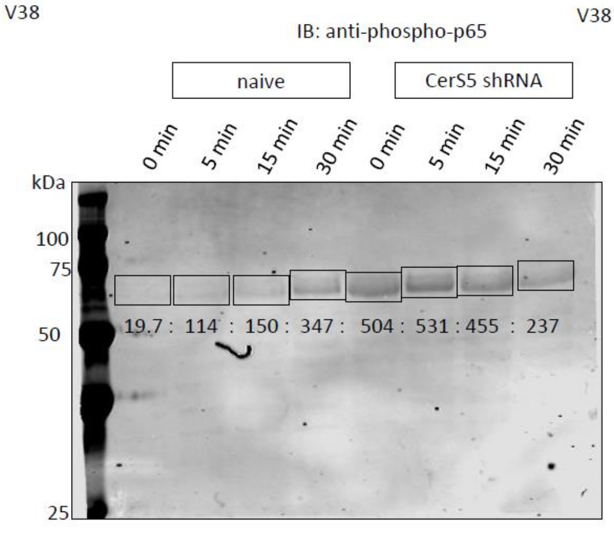
V38
IB: anti-phospho-p65
naive
CerS5 shRNA
0 min 5 min 15 min 30 min 0 min 5 min 15 min 30 min
kDa
100
75
50
25
19.7 : 114 : 150 : 347 : 504 : 531 : 455 : 237

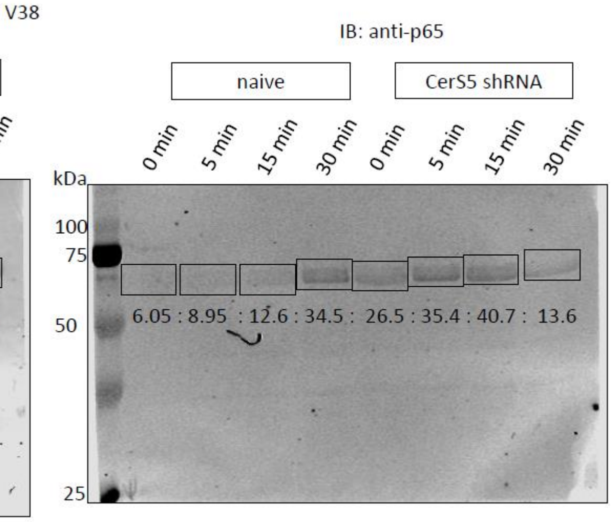
V38
IB: anti-p65
naive
CerS5 shRNA
0 min 5 min 15 min 30 min 0 min 5 min 15 min 30 min
kDa
100
75
50
25
6.05 : 8.95 : 12.6 : 34.5 : 26.5 : 35.4 : 40.7 : 13.6

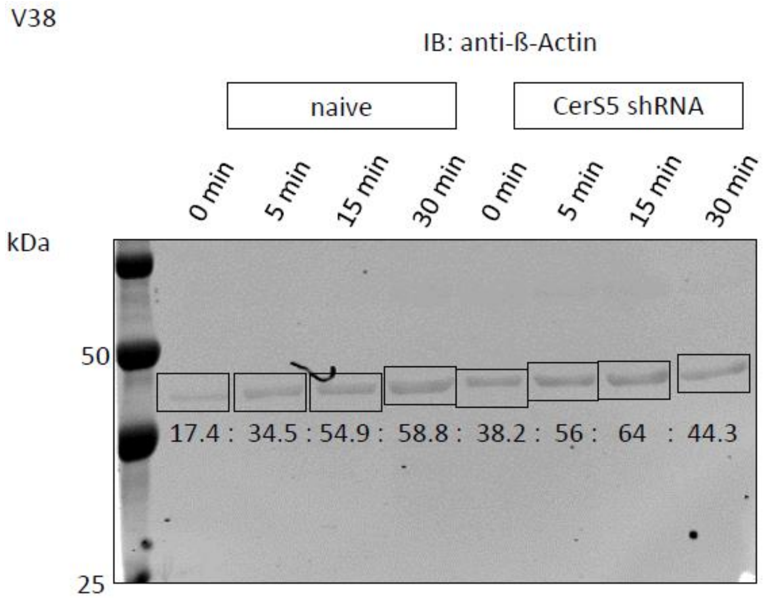
V38
IB: anti-ß-Actin
naive
CerS5 shRNA
0 min
5 min
15 min
30 min
0 min
5 min
15 min
30 min
kDa
50
25
17.4 : 34.5 : 54.9 : 58.8 : 38.2 : 56 : 64 : 44.3

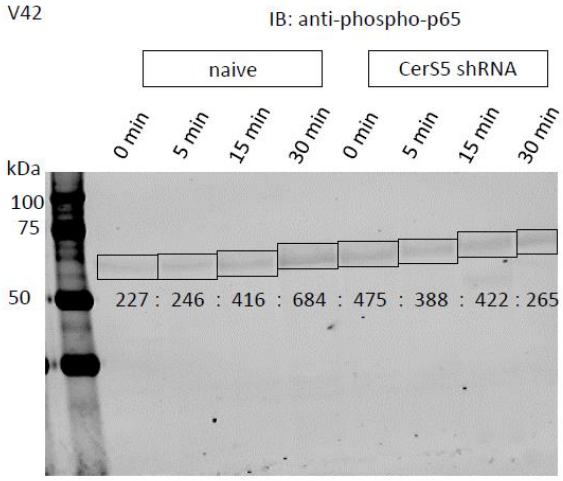
V42
IB: anti-phospho-p65
naive
CerS5 shRNA
0 min
5 min
15 min
30 min
0 min
5 min
15 min
30 min
kDa
100
75
50
227 : 246 : 416 : 684 : 475 : 388 : 422 : 265

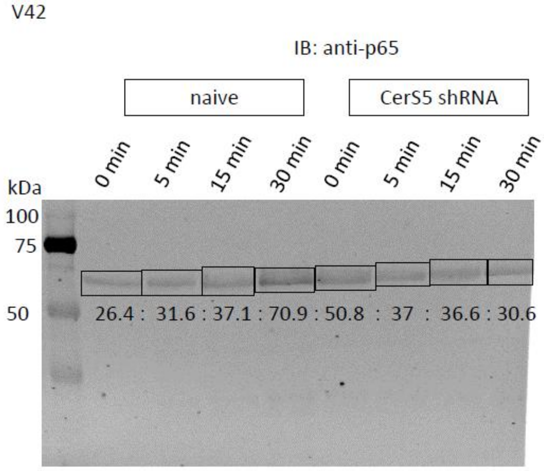
V42
IB: anti-p65
naive
CerS5 shRNA
0 min
5 min
15 min
30 min
0 min
5 min
15 min
30 min
kDa
100
75
50
26.4 : 31.6 : 37.1 : 70.9 : 50.8 : 37 : 36.6 : 30.6

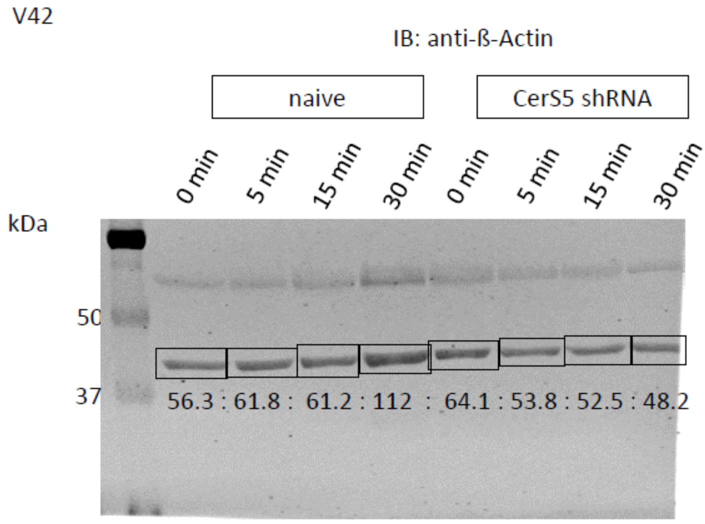
V42
IB: anti-ß-Actin
naive
CerS5 shRNA
0 min
5 min
15 min
30 min
0 min
5 min
15 min
30 min
kDa
50
37
56.3 : 61.8 : 61.2 : 112 : 64.1 : 53.8 : 52.5 : 48.2

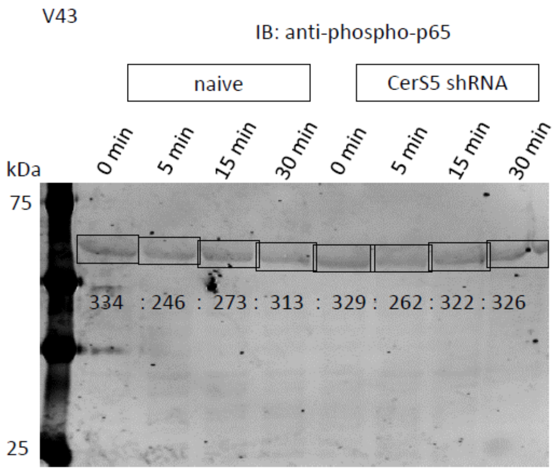
V43
IB: anti-phospho-p65
naive
CerS5 shRNA
0 min
5 min
15 min
30 min
0 min
5 min
15 min
30 min
kDa
75
25
334 : 246 : 273 : 313 : 329 : 262 : 322 : 326

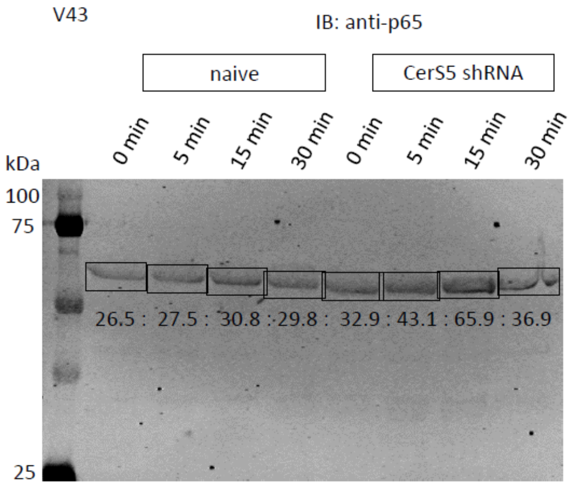
V43
IB: anti-p65
naive
CerS5 shRNA
0 min
5 min
15 min
30 min
0 min
5 min
15 min
30 min
kDa
100
75
25
26.5 : 27.5 : 30.8 : 29.8 : 32.9 : 43.1 : 65.9 : 36.9

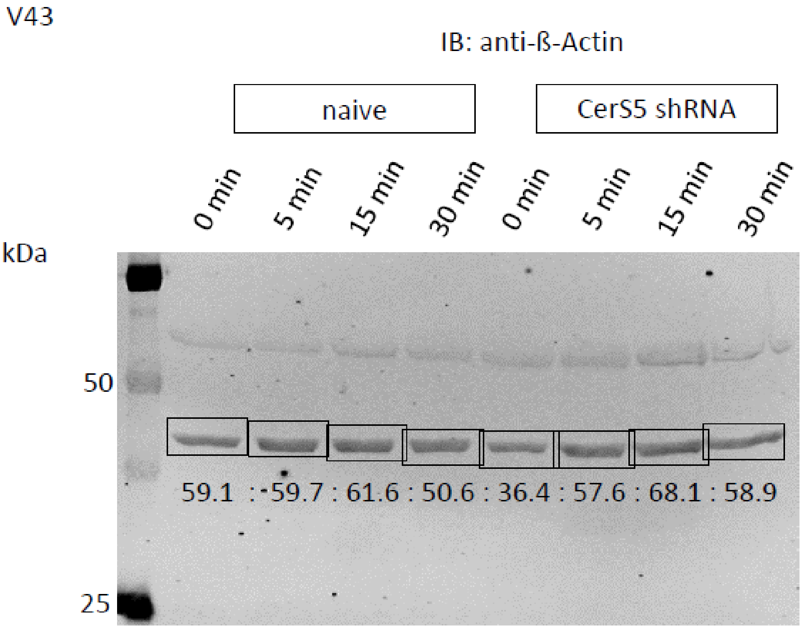
V43
IB: anti-ß-Actin
naive
CerS5 shRNA
0 min
5 min
15 min
30 min
0 min
5 min
15 min
30 min
kDa
50
25
59.1 : 59.7 : 61.6 : 50.6 : 36.4 : 57.6 : 68.1 : 58.9

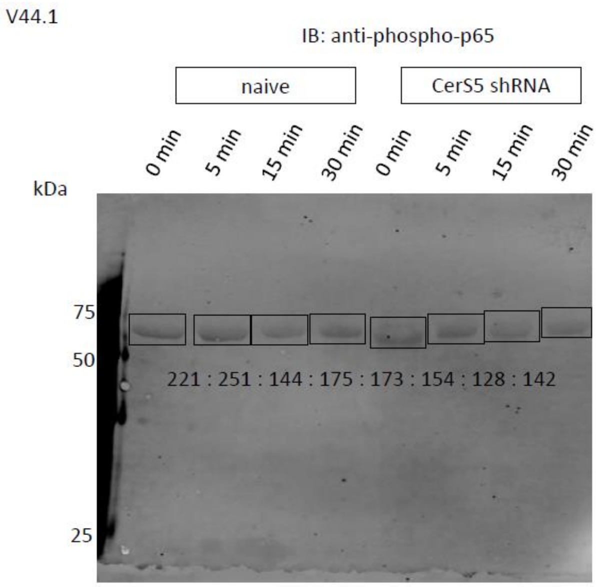
V44.1
IB: anti-phospho-p65
naive
CerS5 shRNA
0 min
5 min
15 min
30 min
0 min
5 min
15 min
30 min
kDa
75
50
25
221 : 251 : 144 : 175 : 173 : 154 : 128 : 142

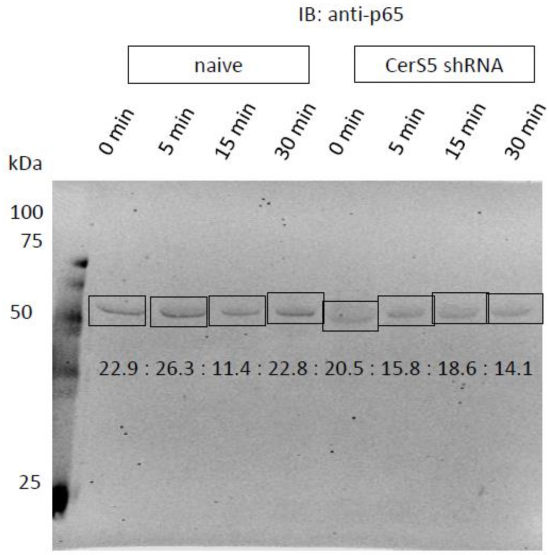
IB: anti-p65
naive
CerS5 shRNA
0 min
5 min
15 min
30 min
0 min
5 min
15 min
30 min
kDa
100
75
50
25
22.9 : 26.3 : 11.4 : 22.8 : 20.5 : 15.8 : 18.6 : 14.1

V44.1

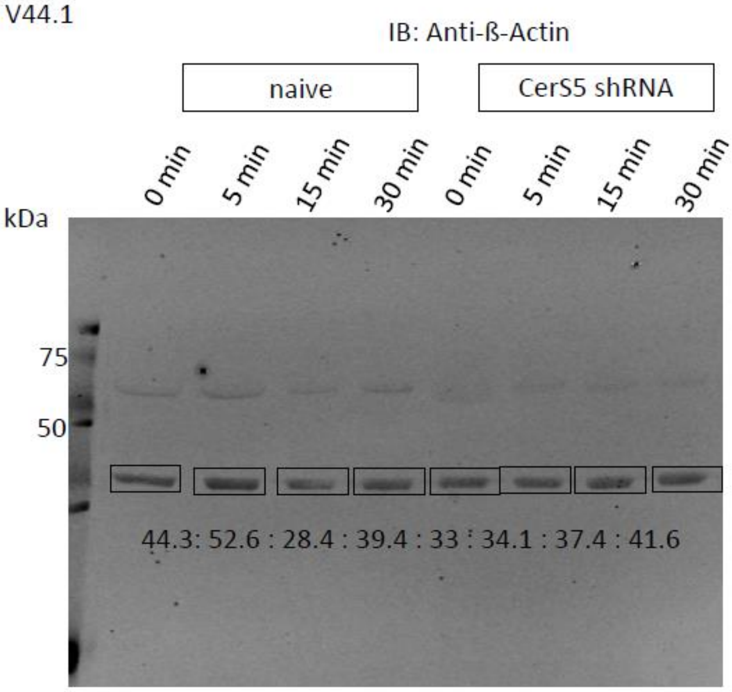
IB: Anti-ß-Actin
naive
CerS5 shRNA
0 min
5 min
15 min
30 min
0 min
5 min
15 min
30 min
kDa
75
50
44.3: 52.6 : 28.4 : 39.4 : 33 : 34.1 : 37.4 : 41.6

V44.2

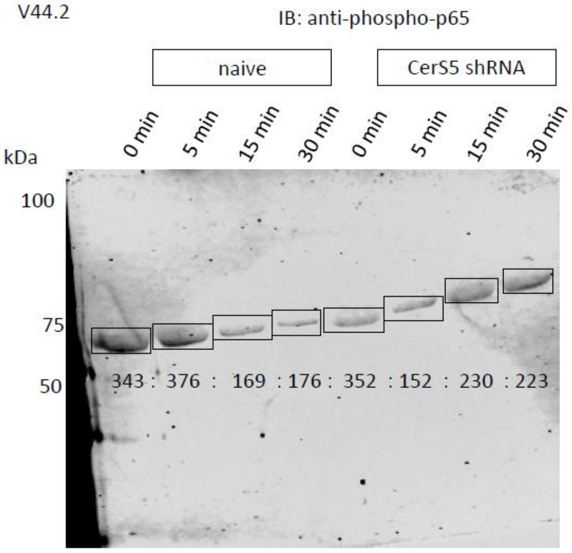
IB: anti-phospho-p65
naive
CerS5 shRNA
0 min
5 min
15 min
30 min
0 min
5 min
15 min
30 min
kDa
100
75
50
343 : 376 : 169 : 176 : 352 : 152 : 230 : 223

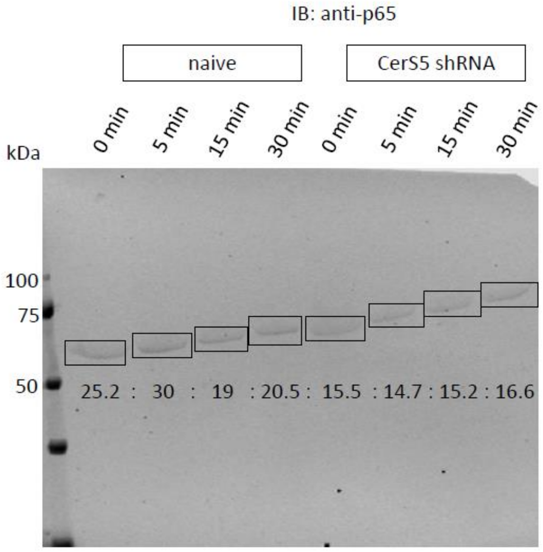
IB: anti-p65
naive
CerS5 shRNA
0 min
5 min
15 min
30 min
0 min
5 min
15 min
30 min
kDa
100
75
50
25.2 : 30 : 19 : 20.5 : 15.5 : 14.7 : 15.2 : 16.6

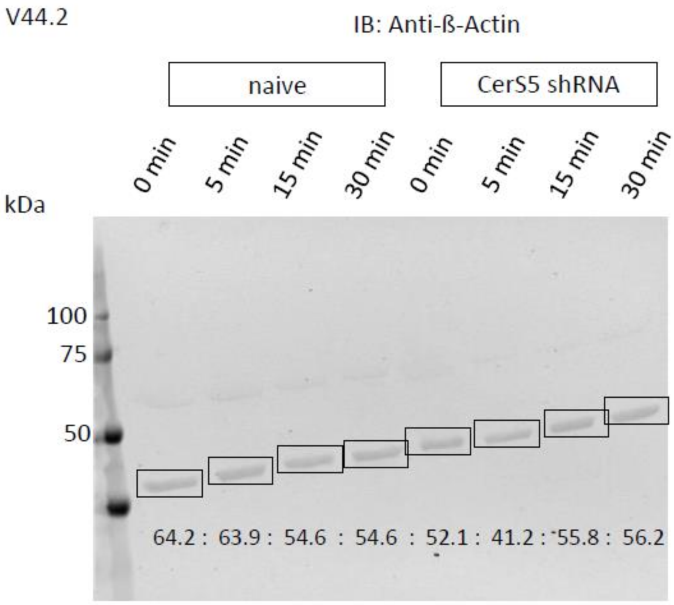

Figure S4. The whole western blot images.
